# Oncogenic function of growth arrest-specific transcript 5 by competing with miR-423-3p to regulate SMARCA4 in hepatocellular carcinoma

**DOI:** 10.1038/s12276-025-01459-4

**Published:** 2025-06-02

**Authors:** Sang Yean Kim, Jin Woong Ha, Min Jeong Na, Soyoung Jeon, Jung Hwan Yoon, Won Sang Park, Suk Woo Nam

**Affiliations:** 1 Department of Pathology, College of Medicine, The Catholic University of Korea, Seocho-gu, Seoul, Republic of Korea; 2 Functional RNomics Research Center, The Catholic University of Korea, Seocho-gu, Seoul, Republic of Korea; 3 NEORNAT Inc., Seocho-gu, seoul, Korea; 4 Department of Biomedicine and Health Sciences, Graduate School, The Catholic University of Korea, Seoul, Korea

**Keywords:** Cancer genomics, Targeted therapies

## Abstract

Long noncoding RNA growth arrest-specific transcript 5 (GAS5) has been identified as a tumor suppressor due to its downregulation in several cancers. However, our comprehensive analyses revealed aberrant overexpression of GAS5 in various cancers, with a direct association with SMARCA4 in hepatocellular carcinoma (HCC). Differential expression analyses were conducted using publicly available transcriptome datasets. Functional studies of GAS5 and its downstream targets in HCC were performed via small interfering RNA-mediated knockdown in various HCC cell lines, in vivo xenograft mouse models and spontaneous liver cancer models in *Ras*-transgenic mice. Here we discover that METTL3-mediated *N*^6^-methyladenosine modification promoted IGF2BP2 binding, stabilizing GAS5 in HCC. GAS5 expression was significantly upregulated in large cohort of patients with solid cancer, including HCC. Targeted disruption of GAS5 resulted in notable inhibition of growth and proliferation in HCC cells. Further analyses demonstrated that GAS5 enhanced in vitro tumorigenesis and metastatic potential of HCC cells. MicroRNA target prediction and functional validation indicated that GAS5 shared a miR-423-3p binding element with SMARCA4 messenger RNA, functioning as a competing endogenous RNA. This interaction was validated in vitro tumorigenesis assays and in vivo models. Moreover, a synergistic effect was observed with vehicle containing a small interfering RNA mixture targeting both GAS5 and SMARCA4 in these animal models. *N*^6^-methyladenosine-mediated IGF2BP2 binding stabilizes GAS5, which functions as a competing endogenous RNA for miR-423-3p, thereby enhancing the translation of SMARCA4 messenger RNA. GAS5 acts as a crucial regulator of the oncogenic SMARCA4 in hepatocellular carcinogenesis, presenting a potential therapeutic target for the treatment of liver malignancies.

## Aberrant GAS5 expression drives liver cancer progression

Hepatocellular carcinoma (HCC) is a common and deadly liver cancer. Despite research, survival rates have not improved much. Scientists are exploring new ways to treat HCC by targeting RNA changes in cancer cells. This study focuses on a molecule GAS5, which is typically low in many cancers but found at elevated levels in HCC. Researchers found that GAS5 helps cancer grow by interacting with other molecules such as miR-423-3p and SMARCA4. They used lab experiments and mouse models to show that reducing GAS5 or SMARCA4 can slow down cancer growth. They also discovered that GAS5 is stabilized by a chemical change called *N*^6^-methyladenosine methylation, which helps it function in cancer cells. By targeting these interactions, the researchers suggest new treatment strategies for HCC. In the future, therapies could focus on disrupting these molecular interactions to improve outcomes for patients with liver cancer. This summary was initially drafted using artificial intelligence, then revised and fact-checked by the author.

## Introduction

Hepatocellular carcinoma (HCC) ranks as the sixth most common cancer globally and is associated with a high mortality rate^1^. Despite extensive research efforts, the overall survival rate for patients with HCC has not significantly improved over the past two decades^2^. Current clinical strategies predominantly focus on antiangiogenesis and enhancing immune responses^3^. Identifying novel molecular targets and underlying mechanisms in HCC is crucial for developing new therapeutic strategies that could improve patient outcomes. Given the emerging significance of aberrant RNA modifications in cancer progression and suppression, targeting RNA-modifying pathways presents a promising approach for liver cancer therapy^4^.

Among the more than 170 identified RNA modifications, RNA methylations are particularly prevalent, accounting for approximately two-thirds of these modifications^5^. Key RNA methylations implicated in tumorigenesis include 2′-*O*-methylation (2′-*O*-Me), *N*^7^-methylguanosine (m^7^G), 5-methylcytidine (m^5^C), *N*^1^-methyladenosine (m^1^A) and *N*^6^-methyladenosine (m^6^A)^6^. Notably, m^6^A modifications play critical roles in regulating RNA stability, transcription, splicing and translation^7^. These processes are mediated by m^6^A-binding proteins (readers), methylating enzymes (writers) and demethylating enzymes (erasers), highlighting the reversible nature of m^6^A modifications and their significant implications for tumorigenesis^4^. A key of methylating enzyme methyltransferase-like 3 (METTL3) is significantly upregulated in HCC and contributes to its progression by repressing SOCS2 (ref. ^8^). Specifically, the insulin-like growth factor 2 messenger RNA-binding proteins (IGF2BPs), including IGF2BP1, IGF2BP2 and IGF2BP3, among the m^6^A-binding proteins function as a family of m^6^A readers that play oncogenic roles in HCC by promoting the accumulation of oncogenic transcripts such as MYC^9^. Previous studies have demonstrated that IGF2BPs, as m^6^A reader, recognize m^6^A-methylated mRNAs by binding to the m6A motif ‘GGAC’. IGF2BPs recruit stabilizing factors to prevent degradation and enhance translation. Consequently, m^6^A-methylated IGF2BP-dependent HCC-specific genes may exhibit increased stability through IGF2BP binding^9^. Accordingly, each component of the m^6^A RNA-modifying machinery could serve as a potential therapeutic target for patients with HCC exhibiting aberrant m^6^A modifications.

Growth arrest-specific transcript 5 (GAS5) is a 651-nucleotide long non-coding RNA (lncRNA) located on chromosome 1q25.1, commonly downregulated in various human cancers, including breast, lung, prostate, colorectal, melanoma, bladder, liver, stomach, cervical, thyroid and ovarian cancers. Traditionally viewed as a tumor suppressor, GAS5 inhibits angiogenesis, proliferation, invasion and migration in multiple malignancies and enhances sensitivity to radiotherapy and chemotherapy by promoting cell cycle arrest^10–12^. GAS5 is also overexpressed in KIRC and HCC, which has been associated with poor overall survival in patients with high GAS5 expression^13,14^. In particular, GAS5 has been identified as a CDKN1-related competing endogenous RNA (ceRNA), with its upregulation driving HCC progression through the GAS5/miR-25-3p/SOX11 network^15^. Recent studies have identified m^6^A modification as a crucial regulator of GAS5, with binding proteins playing significant roles in this process^16^. Integrative analyses of METTL3-, IGF2BPs- and HCC-associated genes using publicly available transcriptome datasets identified GAS5 as a uniquely significant lncRNA in HCC. Our in vitro experiments further confirmed that METTL3-mediated m^6^A modification and IGF2BP2 binding enhance the stability of GAS5 in HCC. Moreover, we observed a significant correlation between GAS5 overexpression and advanced HCC in a large cohort, including multistage HCC transcriptome data. Functionally, GAS5 acts as a ceRNA for miR-423-3p, thereby regulating the mRNA stability and translation of SMARCA4. A subunit of the SWI–SNF complex aberrant overexpression of SMARCA4 contributes to HCC by promoting the IRAK1 enhancer, which facilitates the activation of the oncoproteins Gankyrin and AKR1B10 (ref. ^17^). Therefore, this ceRNA interaction was validated through in vitro tumorigenesis assays and in vivo models, including *Ras*-transgenic spontaneous HCC mice (*Ras*-Tg). Knockout experiments targeting GAS5 or SMARCA4 and overexpression of miR-423-3p demonstrated reduced tumorigenesis, highlighting the oncogenic role of the GAS5–miR-423-3p–SMARCA4 axis in HCC progression. Here, we suggest that GAS5 enhances SMARCA4 mRNA translation by acting as a ceRNA for miR-423-3p, thereby promoting tumorigenesis. These insights into the GAS5–miR-423-3p–SMARCA4 axis provide a potential therapeutic target for the treatment of liver malignancies.

## Materials and methods

### Tissue samples

A total of 41 matched pairs of HCC tissues with their corresponding noncancerous liver biopsy were obtained from National Biobank of Korea. Written informed consent was obtained from each subject according to the Declaration of Helsinki, and the study was approved by the Institutional Review of Board of the Songeui Campus, College of Medicine, the Catholic University of Korea (Institutional Review of Board approval no. MC18TESI0075).

### Mouse liver cancer model

The H-*ras*12V homozygous transgenic (*Ras*-Tg) mice were kindly provided by Dr. Dae-Yeoul Yu (Laboratory of Human Genomics, Korea Research Institute of Bioscience and Biotechnology, Daejeon, Korea)^18^. The male mice spontaneously developed HCC beginning at approximately ~15–18 weeks of age. The mouse livers were collected at 24 weeks of age and processed for experiments. All animal experiments were undertaken in accordance with the National Institutes of Health’s Guide for the Care and Use of Laboratory Animals, with approval of the Animal Experiment Ethics Committee of the Catholic University of Korea College of Medicine.

### In vivo tumorigenesis study

*Ras*-Tg mice were intravenously injected with Invivofectamine 3.0 (Invitrogen) containing 0.25 mg kg^−1^ of Gas5, Smarca4 small interfering RNA (siRNAs) and mmu-miR-423-3p as previously described^19^. The ultrasonography images were taken at 14, 16, 18, 20 and 22 weeks of age with an ultrasound machine (Philips) by the same medical imaging expert each time.

### Xenograft assay

A total of 1 × 10^6^ Hepa1-6 cells of transfected with Gas5, Smarca4 siRNAs and mmu-miR-423-3p mixed with 0.2 ml PBS (pH 7.4) and 20% (v/v) Matrigel (BD Biosciences). The cell suspensions were subcutaneously injected in 5-week-old athymic female Balb/c-nude mice purchased from OrientBio (Seongnam, Korea). The mice were examined once every 2 days for tumor formation at the injection sites. The calculation of tumor volumes were using: 0.5 × length (L) × width squared (W^2^). Each group consisted of five mice, and the tumor growth was quantified by three orthogonal directions using calipers.

### Luciferase activity assay

The cells were seeded in 12-well plates to 50% confluency for transfection. The cells were cotransfected with 100 ng of psiCHECK-2 plasmid and 100 nM of microRNA (miRNA) mimics. After 48 h, the luciferase activities were measured with a dual-luciferase reporter assay system (Promega) in accordance with the manufacturer’s protocol. The Renilla luciferase activity was normalized to firefly luciferase activity.

### Biotin-labeled RNA pull down assay

Hep3B, Huh7, SNU-182 and SNU-449 cells were transfected with Bio-miR-423-3p or Bio-miR-control in two 60 mm dishes. After 48 h of incubation, the cells were collected by Trypsin–EDTA and washed twice with PBS. The cells were resuspended in 0.5 ml of lysis buffer (20 mM Tris (pH 7.5), 100 mM KCl, 5 mM MgCl2, 0.3% IGEPAL CA-630) and then incubated on ice for 30 min. The lysate was isolated by centrifugation at 13,000 rpm for 30 min, and the supernatant was collected. The lysate was added to the strepatavidin-coated magnetic beads (Invitrogen) and incubated and incubated overnight at 4 °C. The beads were washed with lysis buffer five times, and 100 µl of lysis buffer with DNaseI (2 U µl^−1^) was added. After incubation at 37 °C for 10 min, the lysates were centrifuged at 5,000*g* for 5 min, and the supernatant was discarded. Protein kinase K (20 mg ml^−1^) and 1 µl of 10% SDS in 100 µl of lysis buffer were added to the pellet and incubated at 55 °C for 20 min. The RNA bound to the beads (pulldown RNA) or from 10% of the extract (input RNA) was isolated with Trizol reagent (Invitrogen). The levels of GAS5 and SMARCA4 in the Bio-miR-423-3p pulldown were quantified by Quantitative reverse transcription polymerase chain reaction (qRT–PCR). GAPDH was used for normalization^20^.

### Northern blotting

A total of 20 µg of total RNA and 4 µg of RNA marker (Promega) were separated by electrophoresis on 1% agarose-formaldehyde-MOP gels. The RNA was transferred to a biodyne transfer membrane (Pall corporation) via capillary blotting in 20× SSC for 16 h. The RNA was cross-linked to the biodyne membrane by an ultraviolet cross-linker for 1 min. The membrane was incubated in a rotating oven at 65 °C in 10 ml hybridization buffer for 30 min without radioactive probe. The single-strand probes were generated by the incorporation of radioactive dATP into cDNA using a unidirectional thermocycling ration. The probe purification was performed using the Microspin G-50 columns (Cytiva). The probe was added to 10 ml hybridization buffer in an amount of 1–5 million CPM ml^−1^ and incubated overnight at 65 °C in rotating oven. The membrane were washed with wash buffer I (2× SSC, 0.1% SDS) and wash buffer II (0.1× SSC, 0.1% SDS) at 65 °C with 15 min. A phosphor image analyzer (Bio-Rad) was used for imaging^21^.

### ChIRP

A total of 1.5 × 10^7^ cells were used for the chromatin isolation by RNA purification (ChIRP) experiment. The cells were in cross-link 3% formaldehyde for 30 min mixing at room temperature and followed washing with 1× PBS. The cell lysate and probe oligo hybridization for overnight at 37 °C. The next day, the lysate and bead were mixed for 30 min at 37 °C in an oven, and then, the beads were collected using a magnetic stand. The beads were washed with wash buffer, and the protein elution was recovered after the addition of elution buffer and for 20 min rotating at room temperature and 10 min rotating at 65 °C. The final protein samples were separated in SDS–polyacrylamide gel electrophoresis gel for western blot^22^. The GAS5 probe sequence is: probe 1: 5′- AGTCGACTCCTACCTCGAAA-3′, probe 2: 5′- ACCAGGAGCAGAACCATTAA -3′ and probe 3: 5′- CTCCACACAGTGTAGTCAAG -3′.

### RIP assay

The RNA immunoprecipitation (RIP) assay was performed using a RIP assay kit (MBL) in accordance with the manufacturer’s protocol. Briefly, the HCC cell lines were lysed, and 10% of the cell used total RNA isolation. The cell extracts were removed and incubated with 15 μg of antibody for overnight at 4 °C. The RNA was extracted on the beads and amplified with RT–PCR and qRT–PCR. The sequence of genes information is summarized in Supplementary Table 5.

### Statistical analysis

The survival curves were plotted using the Kaplan–Meier product limit method, and significant differences between survival curves were determined using the log-rank test. All experiments were performed at least three times, and all samples were analyzed in triplicate. The results are presented as mean ± standard error of the mean (SEM). The statistical differences between the groups were evaluated by an unpaired two-tailed Student *t*-test using GraphPad 8.0 software (GraphPad Software). The *P* values less than 0.05 were considered statistically significant.

## Results

### m^6^A methylation and IGF2BP2 binding to lncRNA GAS5 enhances stability and life-cycle of RNA in HCC cells

**Fig. 1:**
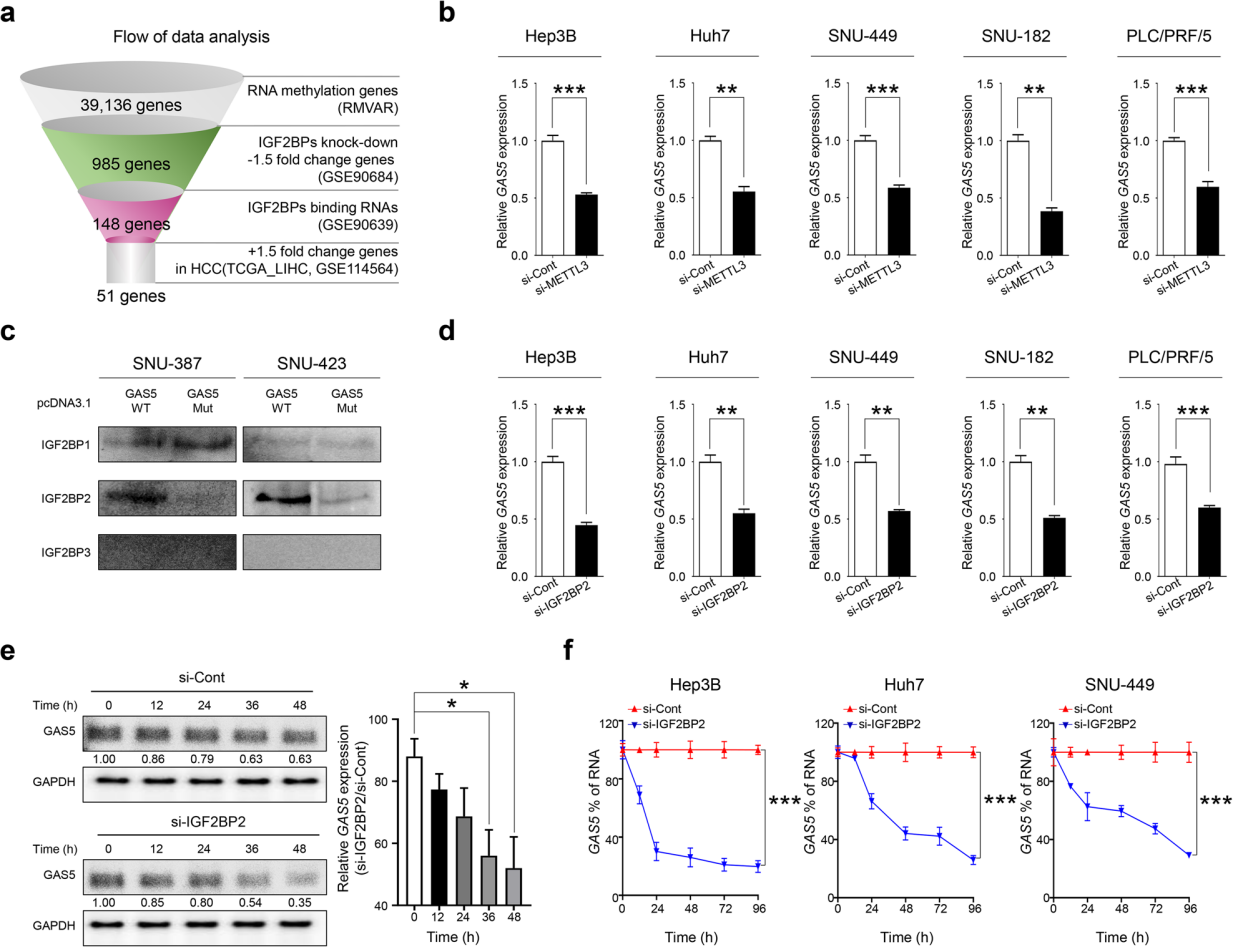
IG2BP2- and METTL3-mediated m^6^A methylation increases GAS5 stability in various HCC cell lines. **a** A schematic strategy image shows how to identify the methylated RNA genes. The Venn diagram shows stabilized genes by the IGF2BP family members, among the overlapping genes of methylated RNA. **b** The expression of GAS5 was measured using qRT–PCR in HCC cell lines, after they were transfected with si-Cont or si-METTL3. **c** The IGF2BP2 binding was determined by ChIRP-WB in HCC cells, after they were transfected with GAS5 WT or GAS5 mutant (Mut) vectors. **d** The expression of GAS5 was measured using qRT–PCR in HCC cell lines, after they were transfected with si-Cont or si-IGF2BP2. **e** The RNA stability was analyzed by northern blot analysis. A bar graph showed the percentage of GAS5 expression of si-Cont- versus si-IGF2BP2-transfected HCC cells. **f** The RNA stability was confirmed by qRT–PCR analysis for GAS5 expression in si-Cont- or si-IGF2BP2-transfected HCC cell lines. GAS5 expression indicates the ratio of si-IGF2BP2 to si-Cont. All the data are shown as the mean ± SEM, *P < 0.05, **P < 0.01, ***P < 0.001; unpaired *t*-test. WT wild type, Mut mutant type.

Recent studies have demonstrated the involvement of global m^6^A modification and IGF2BP proteins in the malignant transformation and growth of various cancer cells^23^. To investigate the role of IGF2BP-associated m^6^A-modified RNAs in HCC, we analyzed publicly available transcriptome datasets (TCGA_LIHC, Catholic_LIHC; GSE114654, ICGC_LIRI, GSE77314). Our analysis revealed that the IGF2BPs family is significantly overexpressed in patients with HCC (≥1.5 fold, *P* < 0.05) and that each IGF2BPs family member is significantly correlated with advanced stages of HCC (Supplementary Fig. 1a, b). Using the RMVAR database (https://rmvar.renlab.org/), we identified 39,136 gene elements as human m^6^A-modified RNA genes. Through gene analysis of the IGF2BPs family knockdown gene signatures, we identified 985 IGF2BPs-dependent m^6^A-modified genes. Subsequent RIP analysis of IGF2BPs narrowed this to 148 genes, and ultimately, we identified 51 genes as aberrantly overexpressed in HCC (Fig. 1a). Notably, among these 51 m^6^A-methylated IGF2BP-dependent HCC-specific genes, GAS5 was the only lncRNA (Supplementary Table 1).

To determine if GAS5 stability requires IGF2BP2- and METTL3-mediated m^6^A modification in HCC cells, we performed RIP with an m^6^A antibody in several HCC cell lines. GAS5 was significantly enriched in all tested cell lines (Hep3B, Huh7, SNU-449, SNU-182 and PLC/PRF/5) (Supplementary Fig. 2a). METTL3 knockdown resulted in reduced GAS5 expression in these cells (Fig. 1b and Supplementary Fig. 2c). Furthermore, RIP with a METTL3 antibody showed enriched binding to GAS5 compared with IgG alone (Supplementary Fig. 2b). We then constructed a wild-type GAS5 expression plasmid (GAS5 WT) and a random mutation form (GAS5 MUT). The GAS5 MUT exhibited no mitotic effects on low GAS5-expressing HCC cells (Supplementary Fig. 3a, b). Although RIP assay with IGF2BP1, IGF2BP2 and IGF2BP3 antibodies and ChIRP assay with GAS5-specific binding probe showed enrichment of IGF2BP1 and IGF2BP2 on GAS5, only IGF2BP2 was observed in substantial amounts (Fig. 1c and Supplementary Fig. 3c–e). IGF2BP2 knockdown reduced GAS5 expression in HCC cells (Fig. 1d and Supplementary Fig. 2d). Northern blot analysis confirmed that IGF2BP2 enhances lncRNA GAS5 stability in HCC cells (Fig. 1e). In addition, GAS5 expression levels were significantly reduced in si-IGF2BP2-transfected HCC cells in a time-dependent manner (Fig. 1f). These results indicate that GAS5 stability is regulated by m^6^A modification and IGF2BP2, positioning them as critical upstream regulators in hepatocellular carcinogenesis.

### Aberrant overexpression of GAS5 in cancers and its contribution to tumorigenic potential in HCC

**Fig. 2:**
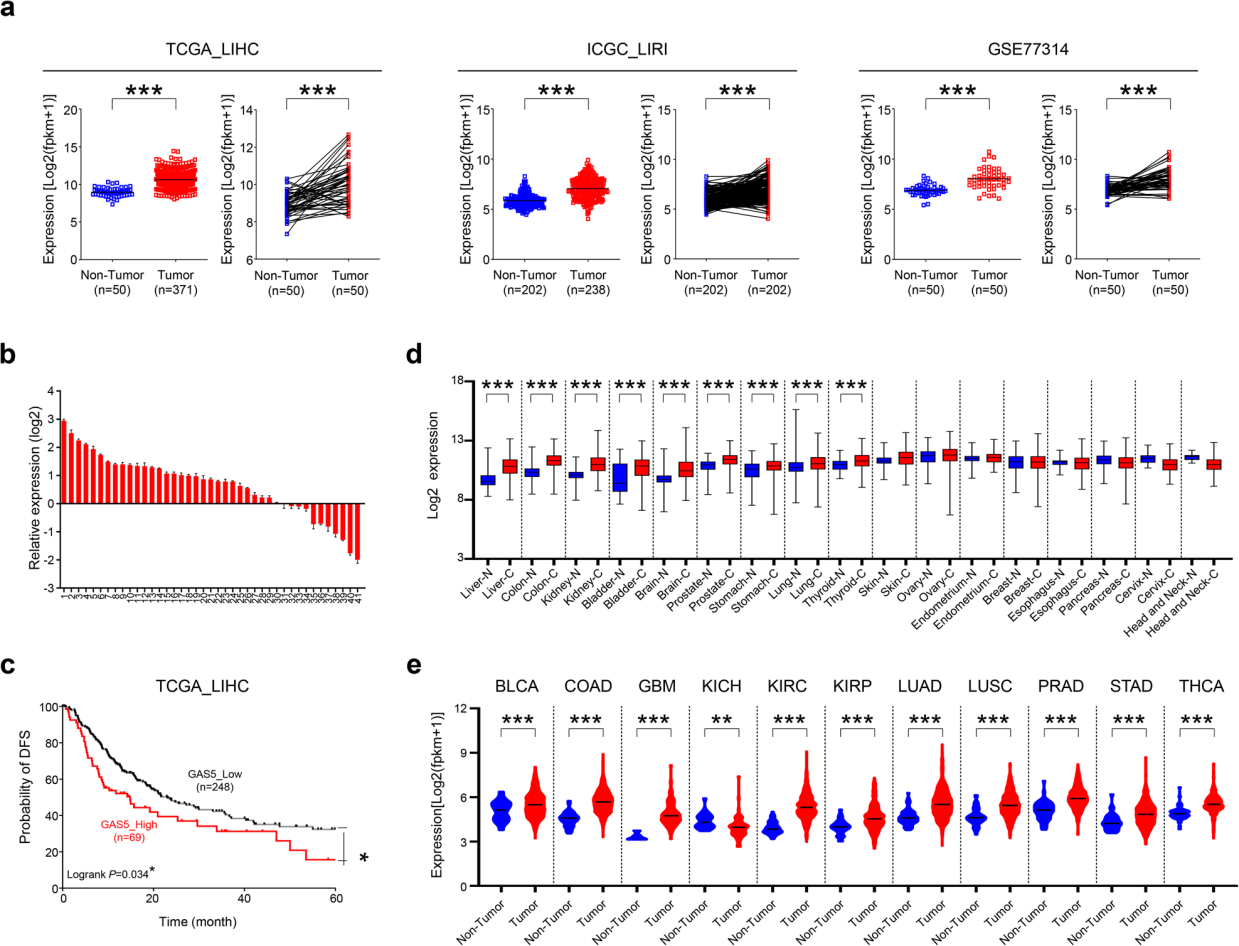
GAS5 overexpression is significantly correlated with both advanced stages and the poor prognosis of many patients with HCC. **a** GAS5 expression in patients with HCC was compared with healthy normal (non-tumor) among three differential datasets (TCGA_LIHC, ICGC_LIRI and GSE77314). **b** The expression of GAS5 in 41 matched pairs of HCC tissues were analyzed by qRT–PCR. **c** The disease-free survival in patients with HCC was compared with GAS5 expression, using Kaplan–Meier survival curves with TCGA_LIHC dataset. **d** A summary of GAS5 expression pattern with fold-changes in 17 different organ-derived tumors in GENT2. **e** The upregulated GAS5 expression pattern in 12 different patients with organ-derived cancer compared with healthy normal (nontumor) in TCGA datasets. All data are shown as the mean ± SEM, *P < 0.05, ***P < 0.001; unpaired *t-*test.

Our observations suggest that m^6^A methylation facilitates IGF2BP2 binding to GAS5, enhancing its stability, and confirm significant overexpression of GAS5 in large cohorts of patients with HCC (Fig. 2a). To validate the aberrant expression of GAS5 in HCC, we conducted quantitative RT–PCR analyses on 41 matched pairs of HCC and corresponding noncancerous liver tissue samples. We found that 25 out of 41 patients with HCC (61%) exhibited significant overexpression of GAS5 in HCC tissues (Fig. 2b). Kaplan–Meier survival analysis of the TCGA_LIHC dataset revealed that patients with HCC with high GAS5 expression had a significantly lower 5-year disease-free survival rate compared with those with low GAS5 levels (Fig. 2c). Further analysis of lncRNA expression patterns in multistage HCC (Catholic_LIHC; GSE114564) showed a gradual increase in GAS5 expression from normal liver tissue through chronic disease and precancer to overt HCC (Supplementary Fig. 4a–c). Previous reports identified GAS5 as a tumor suppressor lncRNA, typically downregulated in cancer cells. To clarify this, we analyzed differential GAS5 expression in cancers using GENT2 and TCGA datasets. Our results showed significant overexpression of GAS5 in 9 out of 17 cancers (GENT2) and 12 out of 34 cancers (TCGA) compared with corresponding noncancerous tissues (Fig. 2d, e). Notably, eight organ-specific cancers exhibited common overexpression of GAS5 across both datasets (Supplementary Table 2)

**Fig. 3:**
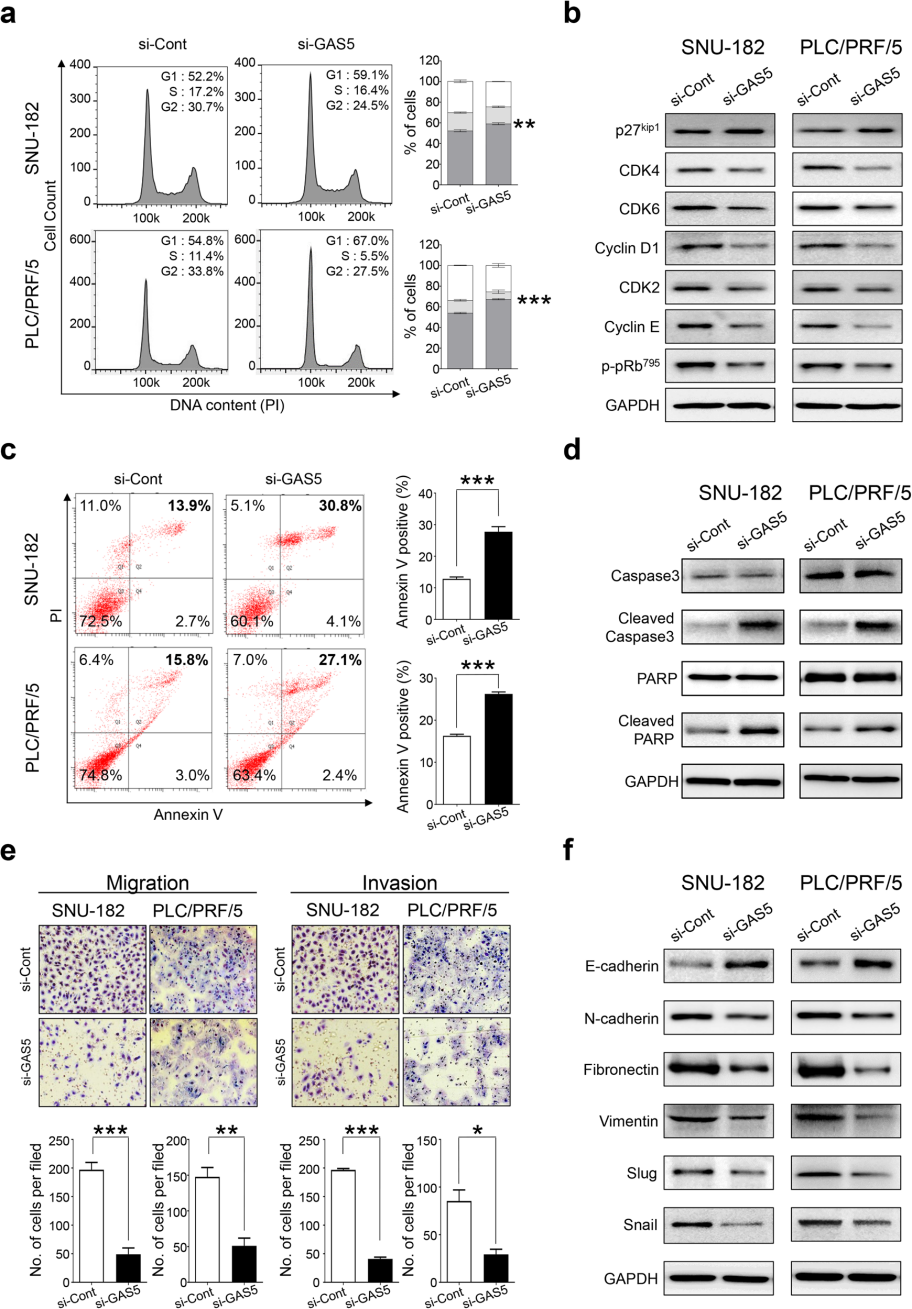
HCC cell lines require GAS5 overexpression for cellular growth, proliferation, and invasive ability. **a** The cell cycle profiles were analyzed after they were transfected with si-Cont or si-GAS5 in SNU-182 and PLC/PRF/5. The bar graph indicates the percentage at each cell cycle phase. **b** The cell cycle modulators were analyzed with western blot analysis in SNU-182 and PLC/PRF/5 transfected with si-Cont or si-GAS5. **c** Annexin-V-FITC/PI-positive SNU-182 and PLC/PRF/5 cells (apoptotic cells) were measured by flow cytometry in SNU-182 and PLC/PRF/5, after they were transfected with si-Cont or si-GAS5. The bar graph indicates the percentage of Annexin-V and PI double positive. **d** The apoptosis modulators were analyzed with western blot analysis in SNU-182 and PLC/PRF/5 transfected with si-Cont or si-GAS5. **e** The Boyden chamber motility assay (left) and transwell invasion assay (right) were evaluated by migrated and invaded cell image (upper) and a bar graph (lower), after they were transfected with si-Cont or si-GAS5 in SNU-182 and PLC/PRF/5. **f** The EMT modulators were analyzed with western blot analysis in SNU-182 and PLC/PRF/5 transfected with si-Cont or si-GAS5. GAPDH was used as a loading control. All the data are shown as the mean ± SEM, *P < 0.05, **P < 0.01, ***P < 0.001; unpaired *t*-test.

To explore the oncogenic functions of GAS5 in hepatocellular carcinogenesis, we conducted in vitro tumorigenesis experiments. We measured GAS5 expression via qRT–PCR in seven HCC cell lines, including immortalized normal hepatic cell lines (MIHA) and selected PLC/PRF/5 and SNU-182 cell lines for further analysis, due to their high GAS5 expression (Supplementary Fig. 5a). Ectopic expression of GAS5-specific siRNA (si-GAS5) significantly suppressed tumor cell growth and proliferation in HCC cells with high GAS5 expression (Supplementary Fig. 5b, c). Flow cytometry analysis of Propidium iodide (PI)-stained cells indicated that GAS5 knockdown induced cell cycle arrest in HCC cells. In addition, GAS5 knockdown selectively suppressed the expression of cell cycle components such as CDK2, CDK4/6, cyclin D1 and cyclin E (Fig. 3a, b and Supplementary Fig. 6a). Flow cytometry of annexin-V-stained cells showed that GAS5 knockdown induced apoptosis, confirmed by increased cleaved caspase-3 and PARP cleavage (Fig. 3c, d and Supplementary Fig. 6b). The epithelial–mesenchymal transition (EMT) is a key process in cancer progression. To investigate the role of GAS5 in the malignant behavior of HCC cells, we performed a scratch wound healing assay. GAS5 knockdown significantly reduced wound healing efficacy and suppressed chemoattractant-stimulated migratory and invasive responses of HCC cells (Fig. 3e and Supplementary Fig. 5d). A western blot analysis of EMT regulatory proteins showed that N-cadherin, Fibronectin, Vimentin, Snail, and Slug were markedly decreased in GAS5 knockdown cells, whereas E-cadherin was increased (Fig. 3f and Supplementary Fig. 6c). Given the significant overexpression of GAS5 in various cancers, we performed growth and proliferation assays on selected cell lines from colon, gastric, glioma, kidney, lung, prostate and thyroid cancers. GAS5 knockdown inhibited cellular growth and proliferation in all tested cancer cell lines with high GAS5 expression (Supplementary Figs. 7–9). These results indicate that aberrant overexpression of GAS5 contributes to the malignant transformation and growth of cancer cells.

### GAS5 interacts Ago2-associated specific miRNAs, miR-423-3p and miR-452-5p

To enhance our understanding of GAS5 function in HCC, we investigated the downstream regulators of GAS5. Previous research has demonstrated that GAS5 can function as a ceRNA to regulate gene expression by sponging miRNAs^24^. We hypothesized that overexpression of GAS5 could bind specific miRNAs, thereby increasing the stability of mRNAs for oncogenic proteins in HCC. Therefore, we explored specific miRNAs that interact with GAS5 in PLC/PRF/5 and SNU-182 HCC cell lines. Before investigating GAS5-associated miRNAs in these cell lines, we examined whether GAS5 can directly interact with the Ago2 protein. Ago2 is a key component of the miRNA-transporting complex crucial for the ceRNA sponge effect^25^. We posited that the interaction between GAS5 and Ago2 could suggest a mechanism for the GAS5-miRNA-mediated sponge effect in HCC. As shown in Supplementary Fig. 10a, Ago2-immunoprecipitations included GAS5, confirmed by RT–PCR in both PLC/PRF/5 and SNU-182 cell lines. This indicates that specific miRNAs are transported to GAS5 via the Ago2-mediated silencing complex in HCC.

**Fig. 4:**
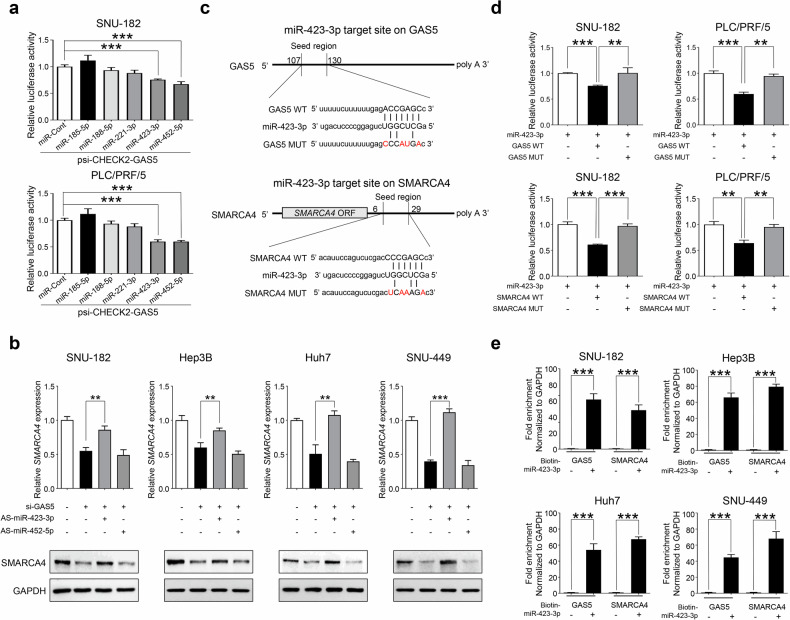
Oncogenic GAS5 sponges miR-423-3p for increased SMARCA4 ceRNA stability in various HCC cell lines. **a** The SNU-182 or PLC/PRF/5 HCC cells were transfected with the psiCheck2-GAS5 vector and sequentially transfected with the indicated miRNAs. Then, the luciferase activity was measured with dual luciferase assays. **b** The relative expression of SMARCA4 was measured with qRT–PCR analysis (top) or western blotting analysis (bottom) in HCC cells, after they were transfected with si-Cont, si-GAS5, AS-miR-423-3p or AS-miR-452-5p. **c** A schematic presentation, the 5′-region of GAS5 and 3′UTR of SMARCA4 have miR-423-3p binding sites. The indicated sequences of either GAS5 (top) and SMARCA4 (bottom) each wild-type (WT) or mutant type (MUT) were inserted in the psiCHECK-2 plasmid. **d** psiCHECK-2 WT or MUT plasmids were transfected into SNU-182 or PLC/PRF/5 HCC cell lines, sequentially transfected with miR-423-3p and measured via dual luciferase activity. **e** The HCC cells were transfected with biotin-labeled microRNA control or biotin-labeled miR-423-3p mimics. The enrichment of GAS5 and SMARCA4 were measured by qRT–PCR, after pulldown with the biotin-labeled miR-423-3p. All the data are shown as the mean ± SEM, **P < 0.01, ***P < 0.001; unpaired *t*-test.

To identify HCC-specific GAS5-binding miRNAs, we employed a combination of database analysis and experimental validation. Using the Encyclopedia of RNA Interactomes (ENCORI) miRNA prediction program (https://starbase.sysu.edu.cn/), we identified 121 miRNAs potentially targeting GAS5. Experimentally, we isolated GAS5-interacting miRNAs using the MS2-binding protein-immunoprecipitation method in both PLC/PRF/5 and SNU-182 cell lines. This approach identified 213 GAS5-interacting miRNAs. A Venn diagram analysis of the 121 predicted miRNAs and the 213 experimentally identified miRNAs revealed 20 GAS5-binding miRNAs. Further refinement using two additional database analyses narrowed this down to five highly GAS5-binding miRNAs. This was achieved through absolute value analysis of 1 in TCGA_LIHC and Catholic_LIHC datasets and commonly overexpressed miRNA analysis in TCGA_LIHC, Catholic_LIHC and Thinghua_LIHC (GSE77276) datasets (Supplementary Fig. 10b). Next, considering that direct interaction between GAS5 and miRNAs can reduce GAS5 stability as part of the ceRNA sponge effect^26^, we performed a dual-luciferase reporter assay. This involved cotransfection of GAS5-luciferase and each of the five miRNA mimics in GAS5-overexpressing PLC/PRF/5 and SNU-182 cell lines. We found that miR-423-3p and miR-452-5p mimics significantly reduced the luciferase activity of GAS5, indicating that these miRNAs specifically bind to GAS5, promoting its degradation in GAS5-overexpressing HCC cells (Fig. 4a). These results suggest that miR-423-3p and miR-452-5p have a direct binding affinity to GAS5 in HCC. Furthermore, we confirmed that the binding regions of miR-423-3p and miR-452-5p are located at sequences 107–130 and 581–602 of GAS5, respectively (Supplementary Fig. 10c).

### Identification of SMARCA4 as potential gene for regulation by sponge effect by GAS5 in HCC

After identifying miR-423-3p and miR-452-5p as functional GAS5-interacting miRNAs in HCC, we investigated potential oncogenic ceRNA candidates regulated by these miRNAs. Our approach involved a three-step database analysis to narrow down the ceRNA candidate genes. First, we used the miRNA target prediction program ENCORI to identify 625 potential target genes for miR-423-3p and 2,614 for miR-452-5p. A Venn diagram analysis revealed 151 overlapping ceRNA candidate genes targeted by both miR-423-3p and miR-452-5p (Supplementary Fig. 11a). Next, considering that overexpression of these candidate genes is necessary for their sponge effects in HCC development, we filtered the list to 36 genes commonly overexpressed in large HCC cohort datasets (TCGA_LIHC, Catholic_LIHC, ICGC_LIRI and GSE77314). Finally, we identified eight ceRNA candidate genes that significantly correlated with the expression pattern of GAS5 in many patients with HCC. Among these, SMARCA4 emerged as the top candidate gene (Supplementary Table 3). Our recent study has reported that SMARCA4 overexpression promotes the upregulation of oncoproteins such as Gankyrin and AKR1B10 through direct transcriptional regulation by IRAK1 in HCC development^17^. We thus explored how SMARCA4 mRNA is stabilized as a ceRNA through interactions between the oncogenic lncRNA GAS5 and specific miRNAs (miR-423-3p or miR-452-5p).

### GAS5 functions as sponge for miR-423-3p to increase SMARCA4 expression in HCC

We conducted an in vitro experimental analysis to understand how the oncogenic GAS5 lncRNA regulates SMARCA4 mRNA as a ceRNA through its interaction with miRNAs (miR-423-3p and miR-452-5p) in HCC cell lines. We examined the sponge effect of GAS5 on these miRNAs in both GAS5-overexpressing HCC cell lines (SNU-182 and Hep3B) and non-GAS5-overexpressing cells (Hep3B, Huh7 and SNU-449). Upon knocking down GAS5 with siRNA, we observed a significant reduction in SMARCA4 expression at both mRNA and protein levels across all tested HCC cell lines, as determined by qRT–PCR and western blot analysis. Since an increased number of miRNAs can abolish the sponge effect of GAS5, we tested whether adding miR-423-3p or miR-452-5p mimics could reduce SMARCA4 expression. Interestingly, only the miR-423-3p mimic significantly reduced SMARCA4 levels in all tested HCC cell lines (Supplementary Fig. 11b, c). In addition, we constructed complementary antisense microRNAs for miR-423-3p (AS-miR-423-3p) to investigate whether the suppression of microRNA expression leads to an increase in SMARCA4 expression. After knocking down GAS5 using siRNA and transfecting with AS-miR-423-3p or AS-miR-452-5p, we observed a significant increase in SMARCA4 levels in all HCC cell lines treated with AS-miR-423-3p, as confirmed by qRT–PCR and western blot analysis (Fig. 4b and Supplementary Fig. 11d). Furthermore, when AS-miR-423-3p or AS-miR-452-5p were transfected into GAS5 knockdown HCC cell lines, we observed that cell growth and proliferation were rescued in the cell lines treated with AS-miR-423-3p (Supplementary Fig. 12). This indicates that the sponge effect between GAS5 and SMARCA4 is regulated by miR-423-3p, not by miR-452-5p.

To further demonstrate that miR-423-3p specifically and directly interacts with the 5′ region of GAS5 and the 3′ region of SMARCA4, we conducted experiments with mutants of miR-423-3p binding sites. We used four cloned luciferase vectors: GAS5 transcript (GAS5 WT), GAS5 with mutated miR-423-3p response sequence (GAS5 MUT), 3′ untranslated region (UTR) of SMARCA4 (SMARCA4 WT), and SMARCA4 with mutated miR-423-3p response sequence (SMARCA4 MUT) (Fig. 4c). Relative luciferase activity was measured after cotransfection with miR-423-3p. miR-423-3p significantly decreased luciferase activity in the WT groups but not in the MUT groups in both SNU-182 and PLC/PRF/5 HCC cell lines (Fig. 4d). This was confirmed through biotin-labeled RNA pulldown assays, where Bio-miR-423-3p mimics enriched GAS5 lncRNA and SMARCA4 mRNA compared with the control (Fig. 4e). In addition, we showed that overexpression of SMARCA4 could be downregulated by increasing miR-423-3p expression. In PLC/PRF/5 cells, which lack SMARCA4, cotransfection of SMARCA4-overexpressing plasmids with miR-423-3p mimics significantly reduced SMARCA4 levels at both mRNA and protein levels, demonstrating the sponge effect (Supplementary Fig. 11e). Collectively, our findings demonstrate that oncogenic GAS5 lncRNA regulates SMARCA4 mRNA expression through direct binding with miR-423-3p in HCC, functioning as a ceRNA sponge.

### Targeted disruptions of GAS5 and SMARCA4 suppresses tumorigenic potential of HCC cells

**Fig. 5:**
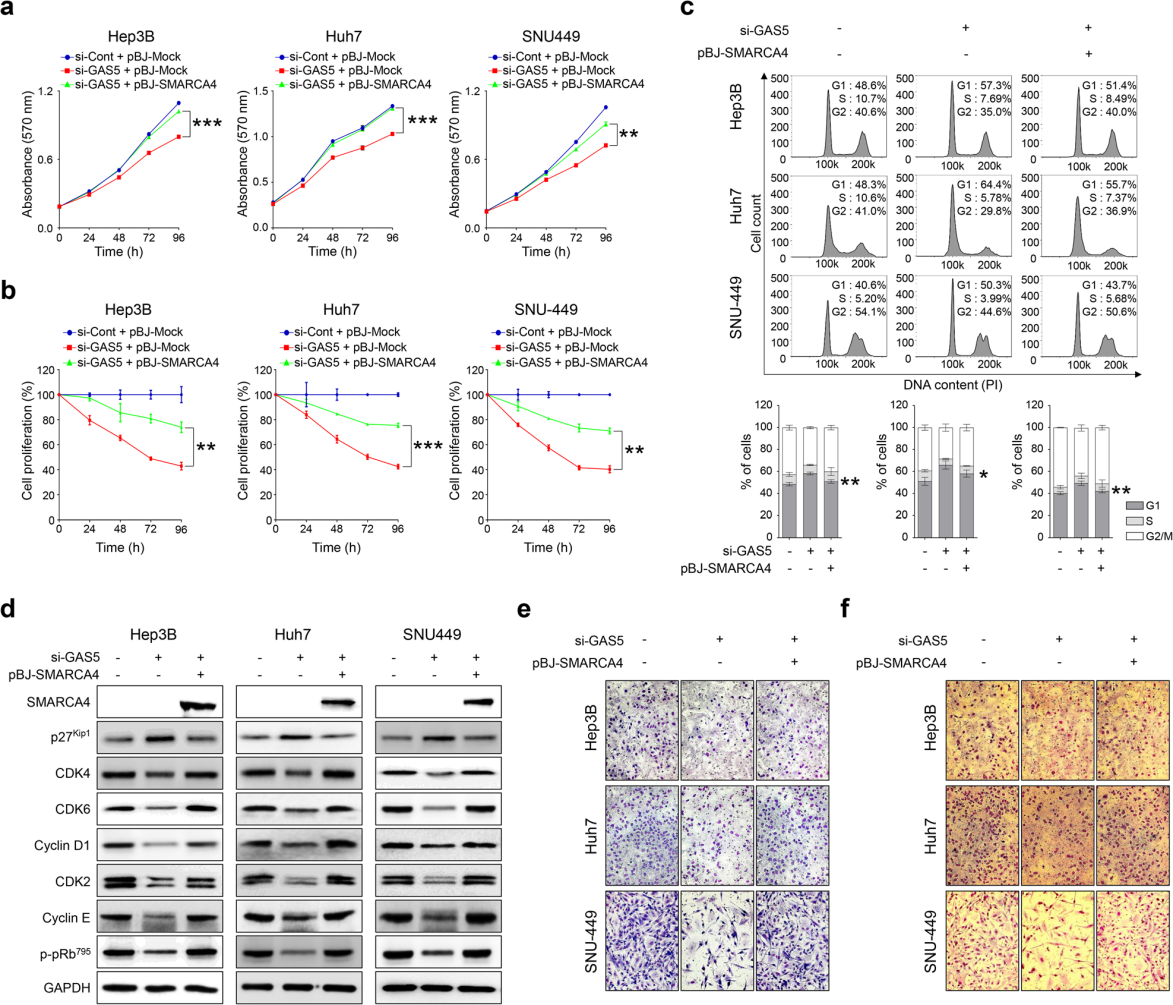
Knockout of GAS5 lncRNA followed by overexpressing SMARC4 mRNA oncogene increase tumorigenesis in HCC cell lines. **a** The cell growth was measured by an MTT assay after transfection of si-Cont and si-GAS5 followed pBJ-SMARCA4 in HCC cells. **b** The cell proliferation was measured by BrdU assay after transfections, as in **a**. **c** Top: the cell cycle profiles were analyzed after transfections equal to **a**. Bottom: the bar graph indicates the percentage at each cell cycle phase in HCC cells. **d** The cell cycle modulators were analyzed with western blot analysis in HCC cells, after they were transfected equal to **a**. GAPDH was used as a loading control. **e**, **f** The Boyden chamber motility assay (**e**) and transwell invasion assay (**f**) were performed with analysis of cell images in HCC cells, after transfection equal to **a**. All the data are shown as the mean ± SEM, *P < 0.05, **P < 0.01, ***P < 0.001; unpaired *t*-test.

In our previous study, knockdown of SMARCA4 with siRNA significantly reduced growth, proliferation and migration in HCC cells^17^. To test whether GAS5 knockdown or miR-423-3p overexpression would produce similar tumorigenic phenotypes as SMARCA4 knockdown, we conducted in vitro analyses using MTT and BrdU assays. Knockdown of GAS5 (si-GAS5), SMARCA4 (si-SMARCA4) or overexpression of miR-423-3p mimics significantly inhibited tumor cell growth and proliferation rates to similar degrees in Hep3B, Huh7 and SNU-449 cell lines (Supplementary Fig. 14a, b). Notably, si-SMARCA4 exhibited a greater inhibition of cellular proliferation over time compared with si-GAS5 or miR-423-3p mimics, suggesting that targeting downstream molecules in the sponge effect could be a more effective strategy for inhibiting HCC development. Flow cytometric analysis of PI-stained DNA showed similar levels of induced G1/S arrest among cells transfected with si-GAS5, si-SMARCA4 or miR-423-3p mimics in all three HCC cell lines (Supplementary Fig. 14c and Supplementary Fig. 15a). We propose that G1/S arrest is a key pathway targeted by GAS5–miR-423-3p–SMARCA4-related sponge effects in HCC. Furthermore, major cell cycle proteins were significantly downregulated to similar levels in all three HCC cell lines transfected with si-GAS5, si-SMARCA4 or miR-423-3p mimics (Supplementary Fig. 14d and Supplementary Fig. 15b). Consistent with previous results (Supplementary Fig. 14a, b), si-SMARCA4-transfected cells exhibited a more pronounced reduction in cell cycle proteins than cells transfected with si-GAS5 or miR-423-3p mimics (Supplementary Fig. 14c and Supplementary Fig. 15a). Moreover, individual transfection with si-GAS5, si-SMARCA4 or miR-423-3p mimics similarly suppressed chemoattractant-stimulated responses in the three HCC cell lines, as determined by serum-stimulated migration and invasion assays (Supplementary Fig. 14e, f). We performed a rescue experiment to confirm that as a downstream molecule of GAS5, SMARCA4 is involved in HCC development. After knockdown of GAS5 with siRNA and cotransfection with pBJ-SMARCA4, we observed that cell growth and proliferation were rescued when SMARCA4 was overexpressed (Fig. 5a, b). Moreover, G1/S arrest was rescued in cell cycle regulation, and the expression of cell cycle proteins was also fully recovered (Fig. 5c, d and Supplementary Fig. 13a). Similarly, in the cell migration and invasion assays, we observed that the results were consistent with the previous rescue experiment, showing restoration (Fig. 5e, f and Supplementary Fig. 13b, c). We confirmed that a downstream molecule of GAS5, SMARCA4 is an important factor in HCC development through the sponge effect of the GAS5–miR-423-3p–SMARCA4 pathway.

### In vivo validation of oncogenic function of GAS5, SMARCA4 in mouse liver cancer models

We next investigated whether inhibiting the oncogenic sponge effect involving GAS5, miR-423-3p and SMARCA4 could serve as a potential strategy for liver cancer intervention. For this purpose, we used *Ras*-transgenic mice that spontaneously develop HCC in mice (Supplementary Fig. 16a)^19^.

**Fig. 6:**
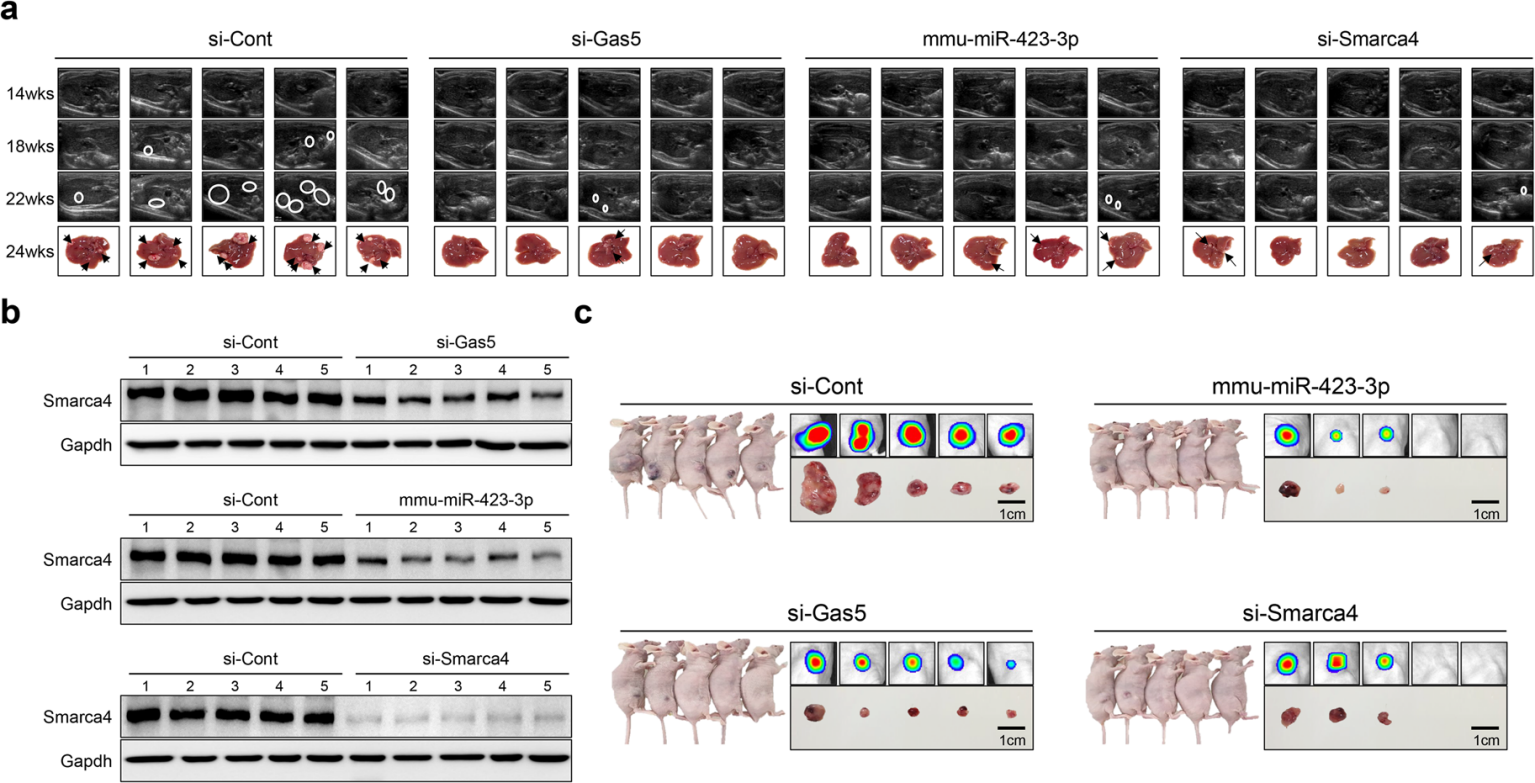
Knockout (GAS5 or SMARCA4) and overexpression (miR-423-3p) similarly repress oncogenic potential ability for both in vivo *Ras*-Tg spontaneous liver cancer and xenograft mouse models. **a** The representative ultrasonography images of *Ras*-Tg mice injected with si-Cont, si-Gas5, mmu-miR-423-3p and si-Smarca4 and liver image at 24 weeks of age. **b** The Smarca4 expression was performed using western blotting analysis with liver tissues from the *Ras*-Tg mouse model. **c** Five mice in each group were subcutaneously injected with Hepa1-6 cells transfected with si-Cont or siRNA specific for the indicated gene and mmu-miR-423-3p mimic. Then, they were observed with mouse images, fluorescence and mass image.

We observed initial HCC detection at 18 weeks in the control siRNA (si-Cont) group, with all five mice developing multiple HCCs by 22 weeks. By contrast, the initial HCC detection occurred at 22 weeks in the si-Gas5, mmu-miR-423-3p and si-Smarca4 groups, with significantly fewer mice developing HCCs by 24 weeks: one of five in the si-GAS5 group, three of five in the mmu-miR-423-3p group and two of five in the si-Smarca4 group (Fig. 6a and Supplementary Fig. 16b). Moreover, the liver-to-body weight ratio was significantly reduced in all treatment groups compared with the si-Cont group (Supplementary Fig. 16c). qRT–PCR and western blot analyses of liver tissues confirmed a direct correlation between reduced Gas5 and Smarca4 expression and liver cancer prophylaxis (Fig. 6b and Supplementary Fig. 16d, e). These findings indicate that inhibiting the oncogenic sponge effect involving GAS5, miR-423-3p and SMARCA4 significantly contributes to liver cancer prevention in vivo.

**Fig. 7:**
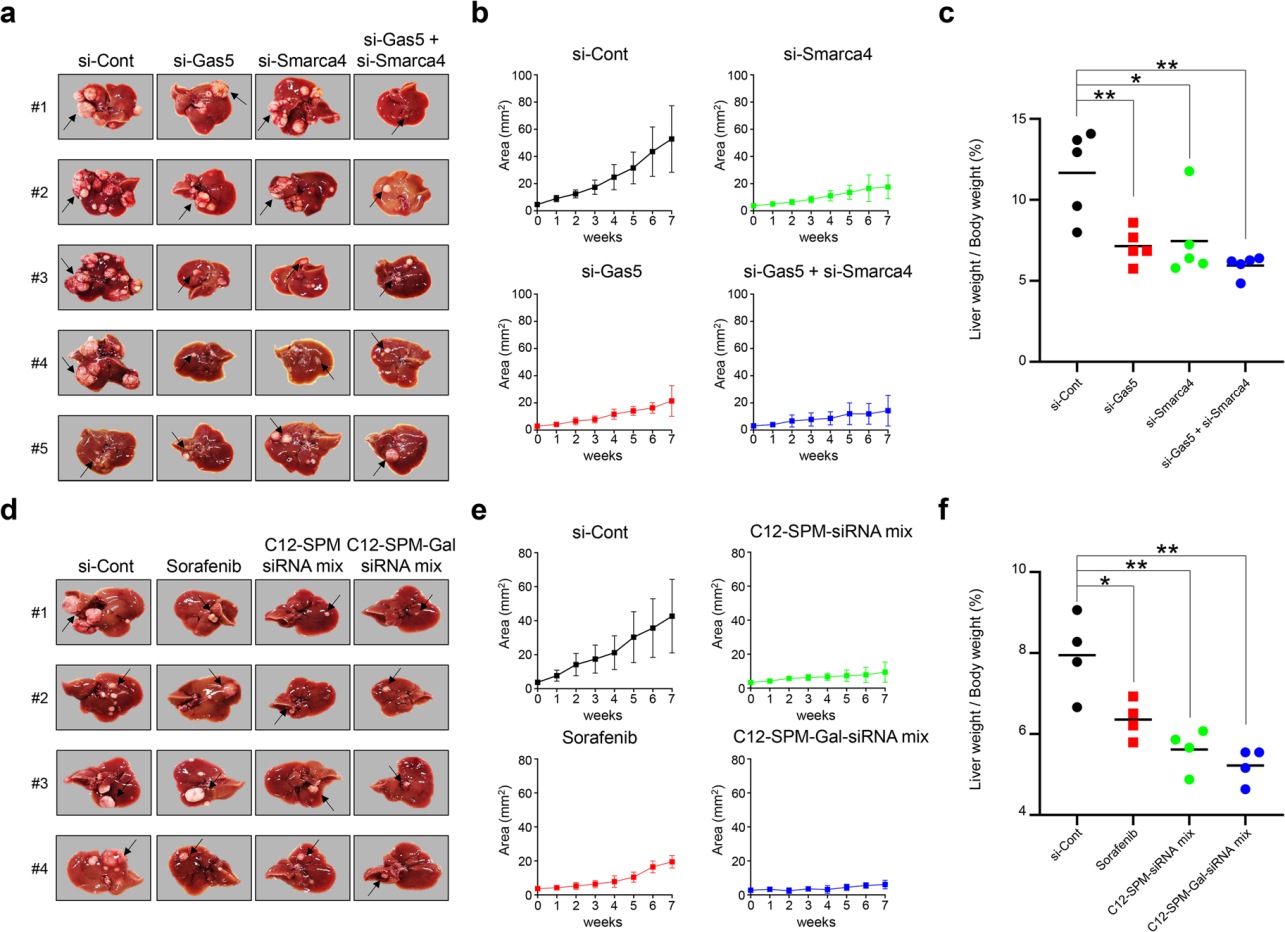
Single or double knockout of GAS5 and SMARCA4 oncogenes significantly inhibit tumorigenesis in liver cancer models in vivo, as being highly evaluated for therapeutic targets. **a** The liver images were analyzed with *Ras*-Tg mice at 8 weeks after injection of si-Cont, si-Gas5, si-Smarca4 or si-Gas5 + si-Smarca4 mix in *Ras*-Tg mouse models. **b** The graph shows the liver tumor area in each group. **c** The dot plots show liver weight per body weight ratio in each group. **d** The liver images were analyzed with *Ras*-Tg mice at 8 weeks after injection of si-Cont, sorafenib, C12-SPM-siRNA mix or C12-SPM-GAL-siRNA combination in *Ras*-Tg mouse models. **e** The graph shows the liver tumor area in each group. **f** The dot plots show the liver weight per body weight ratio in each group. All the data are shown as the mean ± SEM, *P < 0.05, **P < 0.01; unpaired *t*-test.

To further validate these results, we used an in vivo xenograft mouse model. The mice were subcutaneously injected with Hepa1-6 cells transfected with si-Gas5, mmu-miR-423-3p or si-Smarca4. This confirmed significant inhibition of oncogenic effects in HCC development (Fig. 6c and Supplementary Fig. 16f, g). Next, we tested the therapeutic effect of inhibiting the oncogenic sponge effect in *Ras*-Tg spontaneous liver cancer models. Once tumors were confirmed by ultrasonography, we injected si-Gas5, si-Smarca4 and a si-Gas5 + si-Smarca4 mix into the tail vein weekly for 8 weeks (Supplementary Fig. 17a). The tumor growth was monitored weekly via ultrasonography, and liver cancers were confirmed when the mice were killed at 8 weeks post onset. The mice in the control group developed multiple large tumors, while smaller tumors were observed in the si-Gas5, si-Smarca4 and si-Gas5 + si-Smarca4 groups (Fig. 7a). A detailed analysis showed that the si-Gas5 + si-Smarca4 mix inhibited tumor growth more effectively than either si-Gas5 or si-Smarca4 alone (Fig. 7b, c and Supplementary Fig. 17b, c).

Next, we encapsulated the si-Gas5 + si-Smarca4 mix into C12-SPM and C12-SPM-GAL liposomal nanoparticles for liver-specific delivery. qRT–PCR and western blot analyses confirmed the knockdown efficiency of these nanoparticles compared with lipofectamine in Hep3B HCC cells (Supplementary Fig. 18a, b). Similarly to the previous therapeutic effect evaluation model, we confirmed liver cancer development and injected the C12-SPM-siRNA mix or C12-SPM-GAL-siRNA mix weekly into the tail vein, with intraperitoneal sorafenib serving as a positive control. The mice treated with the siRNA mixes or sorafenib showed significantly smaller tumors compared with the control group (Fig. 7d). The C12-SPM-GAL-siRNA mix group showed the most pronounced inhibition of tumor growth (Fig. 7e, f).

## Discussion

In our study, we focused on the GAS5 due to its increased expression in advanced HCC. Contrary to its established role as a tumor suppressor in other cancers^27^, our data indicate that GAS5 may function as an oncogene in HCC. An analysis of multiple transcriptome datasets revealed that GAS5 is significantly overexpressed in advanced HCC compared with early stages and noncancerous tissues. Moreover, higher GAS5 levels correlate with poor survival rates in patients with HCC. Functional assays using GAS5 siRNA in HCC cell lines demonstrated that reducing GAS5 expression significantly inhibited tumor growth, proliferation, survival and metastasis. Moreover, GAS5 knockdown induced apoptosis and cell cycle arrest at the G1/S phase, indicating its essential role in HCC tumorigenesis. These findings suggest that targeting GAS5 could be a potential therapeutic strategy for HCC. We have verified the therapeutic potential of targeting GAS5 and SMARCA4 in HCC with our results (Fig. 7). However, it is commonly known that GAS5 may also function as a tumor suppressor gene. Its role could be context dependent, exhibiting either tumor-suppressive or protumorigenic effects depending on the cellular environment. The GAS5-targeted therapeutic strategy we propose may be specific to HCC. We suggest that in environments where the upregulation of GAS5 acts as a ceRNA sponging miR-423-3p to enhance the expression of the oncogene SMARCA4, targeting GAS5 could be a promising new therapeutic approach.

The oncogenic potential of GAS5 may be partly attributed to its m^6^A RNA modification, which increases its stability. m^6^A is a prevalent and dynamic RNA modification that plays a crucial role in various cellular processes, including RNA stability, splicing and translation^23^. m^6^A modification is regulated by a complex machinery of ‘writers’, ‘readers’ and ‘erasers’. Writers, such as the methyltransferase METTL3, add the m^6^A mark, while readers, such as IGF2BP2, recognize and bind to m^6^A-modified RNAs, influencing their fate^28^. Erasers, such as FTO and ALKBH5, remove the m^6^A marks. Our data show that the m^6^A modification of GAS5, mediated by METTL3, is crucial for its overexpression in HCC. METTL3 has been shown to have oncogenic properties in liver cancer. It promotes tumorigenesis by enhancing the stability and translation of m^6^A-modified oncogenic mRNAs and lncRNAs, thus facilitating their accumulation and function^28^. In the context of HCC, METTL3 overexpression has been linked to poor prognosis and increased tumor growth^4^. By methylating specific target RNAs, METTL3 can modulate key signaling pathways involved in cell proliferation, migration and invasion.

The involvement of m^6^A reader protein IGF2BP2 further supports the role of this modification in stabilizing GAS5. IGF2BP2 recognizes and binds to m^6^A-modified RNAs, protecting them from degradation (Fig. 1e, f). This interaction enhances the stability and longevity of these RNAs, allowing them to exert their oncogenic effects more effectively. In HCC, the binding of IGF2BP2 to m^6^A-modified GAS5 prevents its degradation, leading to its accumulation and promoting tumorigenesis. Disruption of this m^6^A modification pathway could therefore reduce GAS5 levels and inhibit tumor progression. Targeting METTL3 or IGF2BP2, either through genetic knockdown or pharmacological inhibition, could decrease the stability of GAS5, leading to reduced expression of this oncogenic lncRNA and subsequent inhibition of HCC growth.

Furthermore, we explored the ceRNA network involving GAS5. We found that GAS5 acts as a sponge for miR-423-3p, thereby regulating the expression of SMARCA4, an oncogene in HCC. miR-423-3p typically suppresses SMARCA4, but the binding of GAS5 to miR-423-3p releases this suppression, leading to increased SMARCA4 levels. This GAS5–miR-423-3p–SMARCA4 axis promotes HCC progression and disrupting this interaction could provide a novel therapeutic approach. miR-423-3p is a microRNA with dual roles in various cancers, acting as either an oncogene or a tumor suppressor depending on the cellular context. In liver cancer, miR-423-3p has been shown to function predominantly as a tumor suppressor. It exerts its effects by targeting and downregulating oncogenes and other molecules involved in cell proliferation, survival and metastasis^29^. In HCC, miR-423-3p directly targets SMARCA4, a member of the SWI–SNF chromatin remodeling complex, which plays a critical role in gene expression regulation, DNA repair, and cell differentiation^30^. SMARCA4 has been implicated in promoting oncogenic processes such as cell proliferation and resistance to apoptosis in liver cancer^31^. By binding to the 3′UTR of SMARCA4 mRNA, miR-423-3p suppresses its translation and reduces its oncogenic activity. However, the interaction of GAS5 with miR-423-3p disrupts this regulatory mechanism. GAS5 acts as a molecular sponge, sequestering miR-423-3p and preventing it from binding to SMARCA4 mRNA. This sequestration releases the suppression on SMARCA4, leading to its overexpression and the promotion of oncogenic pathways in HCC. As a result, the GAS5–miR-423-3p–SMARCA4 axis becomes a critical driver of HCC progression. Given the central role of miR-423-3p in suppressing SMARCA4, restoring its function or disrupting its interaction with GAS5 could serve as an effective therapeutic strategy. In vivo studies using the *Ras*-transgenic mouse model of HCC demonstrated that silencing GAS5 or SMARCA4 or overexpressing miR-423-3p significantly reduced tumor development. Combining siRNAs targeting both GAS5 and SMARCA4 had a synergistic therapeutic effect, further supporting the potential of this dual-targeting strategy (Fig. 7). Approaches such as miR-423-3p mimics or small molecules that inhibit the ability of GAS5 to sponge miR-423-3p could potentially restore the tumor-suppressive activity of miR-423-3p and reduce SMARCA4 levels, thereby inhibiting HCC growth and progression. This highlights the therapeutic potential of targeting the ceRNA network involving GAS5, miR-423-3p and SMARCA4 in liver cancer. The development of siRNA-based therapeutics targeting GAS5 and SMARCA4 for HCC treatment must be approached with caution. The GAS5–miR-423-3p–SMARCA4 ceRNA mechanism has been specifically identified in HCC, necessitating liver-specific delivery for effective treatment. Recent studies have demonstrated that various lipid nanoparticles (LNPs) can be engineered for efficient hepatic delivery, with modifications permitting targeting to other organs as well^32^. The proposed targeted therapy for GAS5 and SMARCA4 requires the use of liver-specific delivery vehicles, with *N*-acetylgalactosamine (GalNAc) serving as a potential candidate^33^. Which is exclusively expressed in hepatocytes, GalNAc is a ligand for the Asialoglycoprotein Receptor (ASGPR) facilitating selective liver delivery. Several therapeutics utilizing GalNAc have already been developed for this purpose^34^. Furthermore, recent findings indicate that GalNAc-conjugated LNPs are more efficiently delivered to the liver, suggesting that this approach could enhance the therapeutic potential of the targeted siRNA-based treatment^35^. In addition, therapeutics targeting GAS5 and SMARCA4 face challenges related to RNA stability. RNA molecules are inherently unstable in vivo, necessitating modifications to enhance their stability. Previous studies have shown that various modifications, such as 2′-*O*-methoxyethyl (2′-MOE), 2′-*O*-Me, locked nucleic acid and so on and phosphorothioate backbone modifications, can improve RNA stability^36^. Future research will need to address both liver-specific delivery and RNA stabilization challenges, potentially paving the way for the development of new therapeutic strategies for HCC.

**Fig. 8:**
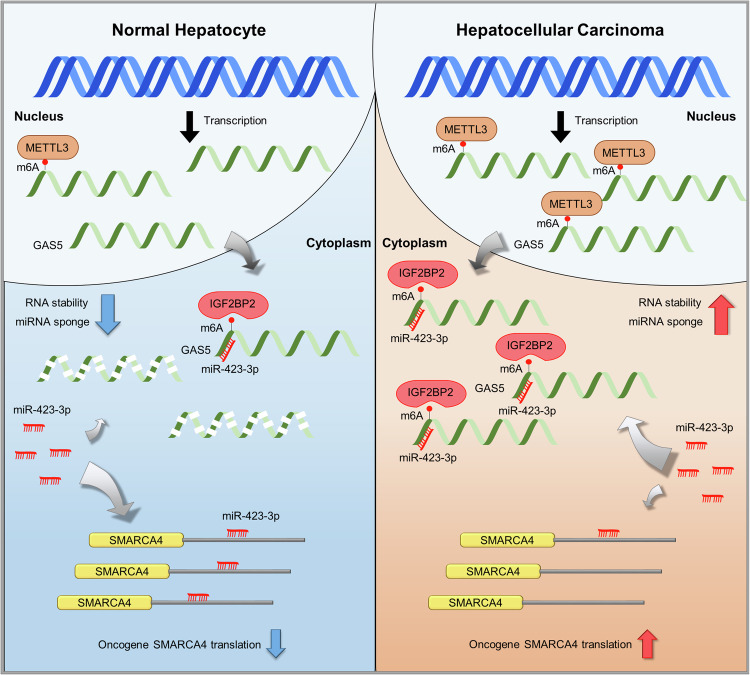
Schematic diagrams that GAS5 sponges miR-423-3p for increased SMARCA4 ceRNA stability in HCC. The proposed schematic model in this study shows that m^6^A-methylated GAS5 is stabilized by IGF2BP2 and sponged miR-423-3p to increase the expression of SMARCA4.

Interestingly, GAS5 overexpression was also observed in several other cancers, including colon, kidney, bladder, brain and prostate cancers. This suggests a broader role for GAS5 in tumorigenesis beyond HCC, possibly through similar m^6^A modification and ceRNA mechanisms. In liver cancer, the role of GAS5 in carcinogenesis is particularly significant. In the context of liver cancer, GAS5 acts through a ceRNA mechanism, where it sponges miR-423-3p. miR-423-3p normally functions as a tumor suppressor by targeting SMARCA4, an oncogene involved in chromatin remodeling and transcriptional regulation. By sequestering miR-423-3p, GAS5 prevents the downregulation of SMARCA4, resulting in increased levels of SMARCA4 and enhanced oncogenic signaling. This GAS5–miR-423-3p–SMARCA4 axis significantly contributes to the aggressive nature of HCC (Fig. 8).

In summary, GAS5 plays a multifaceted role in liver cancer carcinogenesis. Its m^6^A modification and interaction with the ceRNA network underline its importance in promoting tumor growth and survival. Targeting the GAS5–miR-423-3p–SMARCA4 axis presents a novel and promising approach for HCC treatment, potentially improving patient outcomes by disrupting this critical oncogenic pathway.

## Supplementary information

Supplementary Information
