# Supplementary material for: Oncogenic function of growth arrest-specific transcript 5 by competing with miR-423-3p to regulate SMARCA4 in hepatocellular carcinoma
Source: Exp Mol Med. 2025 Jun 2;57(6):1164–76. doi: 10.1038/s12276-025-01459-4 (PMC12229648; doi:10.1038/s12276-025-01459-4)
Supplement: Supplementary file 1 — Supplementary Information [file 12276_2025_1459_MOESM1_ESM.pdf]

Supplementary Information for

Oncogenic Function of Growth Arrest-specific Transcript 5 by Competing with miR-423-3p to Regulate SMARCA4 in Hepatocellular Carcinoma

This file includes:

Supplementary Materials and methods

Supplementary Tables 1 to 5

Supplementary Figures 1 to 18

Supplementary Materials and methods

Publicly available genomic data analyses

To investigate the gene expression levels of subunit genes in HCC, data were obtained from The Cancer Genome Atlas liver hepatocellular carcinoma project (TCGA_LIHC), the International Cancer Genome Consortium Liver Cancer–RIKEN, JP (ICGC_LIRI) and the Gene Expression Omnibus (GEO) database of the National Center for Biotechnology Information (NCBI) (Accession Numbers: GSE77276, GSE77314, GSE89377, and GSE114564). Level 3 mRNA expression data of TCGA datasets HTSeq-FPKM were log2 transformed [log2(fpkm+1)] and used to assess the gene expression levels. GAS5 expression in a variety of cancer was from the Gene Expression database of Normal and Tumor tissues (GENT2, http://gent2.appex.kr/gent2/) and Genomic Data Common (GDC) platform from TCGA datasets.

Cell culture

Human HCC cell lines (Hep3B, HepG2, Huh7, PLC/PRF/5, SNU-182, SNU-354, SNU-368, SNU-423, SNU-449, and SNU-475) were obtained from Korean Cell Line Bank (KCLB, Seoul, Korea). MIHA immortalized HCC cell lines were kindly provided by Dr. Jayanta Roy-Chowdhury (Albert Einstein College of Medicine, New York, NY). Each cell line was cultured in EMEM (ATCC), RPMI-1640 or DMEM medium (GenDEPOT, Katy, TX, USA) containing 10% fetal bovine serum (FBS; GenDEPOT) and 100 units/mL of penicillin-streptomycin (GenDEPOT). All cells were cultured at 37°C in a humidified incubator with 5% CO_2.

Transfection

Small interfering RNAs (si-RNAs) and miRNA mimics were synthesized by Genolution (Seoul, Korea). The sequence of the si-GAS5: 5’- GCAAAGGACUCAGAAUUCA -3’, si-SMARCA4: 5’- CUCGUUCCAGAGCUGAGAU -3’, si-Gas5: 5’- GGCACUGCAAACACAAUGAUU -3’ and si-Smarca4: 5’- CCCGCAUCACUGAGAAGUUUU -3’. SMARCA4 protein expression plasmid, subcloning gene ORF and 3’UTR region (NM_003072) in pBJ plasmid, was purchased from Addgene (Watertown, MA, USA). Transfections were carried out using Lipofectamine RNAiMAX or Lipofectamine 2000 reagent (Invitrogen, Carlsbad, CA, USA), according to the manufacturer’s instructions.

RNA extraction and quantitative real-time polymerase chain reaction (qRT-PCR)

Total RNA was isolated from frozen human tissues and cell lines using TRIzol reagent (Invitrogen, Carlsbad, CA) according to the manufacturer's instruction. For synthesis of cDNAs specific to miRNAs, miScript II RT kit (Qiagen, Manchester, UK) was used. For synthesis of cDNAs specific to mRNAs and lncRNAs, Tetro cDNA synthesis kit (Bioline, London, UK) was used. qRT-PCR was performed with a SensiFAST^{TM} SYBR® NoROX Kit (Bioline). To normalize differences in the amount of total cDNA added to each reaction; U6 for miRNA and glyceraldehyde-3-phosphate dehydrogenase (GAPDH) for mRNA were used as the endogenous control.

Western blotting analysis

Patient frozen tissue (or mouse HCC tissues) and human HCC cell lines were lysed in protein extraction buffer (50mM of HEPES, 5 mM of ethylenediaminetetraacetic acid, 50 mM of NaCl, 1% Triton X-100, 50 mM of NaF, 10 mM of $Na_2P_2O_7$, 1 mM of Na_3VO_4, and 100X Halt protease inhibitor cocktail or 100X Halt protease & phosphatase inhibitor). Lysates containing equal amounts of proteins were separated by sodium dodecyl sulfate/polyacrylamide gel electrophoresis and transferred onto

polyvinylidene difluoride membrane (Bio-Rad, Hercules, CA). The blots were blocked with a 5% skim milk solution and incubated with the respective antibodies information is summarized in Supplementary Table 4. The Immobilon western detection system (Merck Millipore, Burlington, MA) was used to detect antibodies. The Immobilon™ western blot detection system (Millipore) was used to detect bound antibodies. The intensities of the western blot bands were quantified using LAS-4000 (Fuji Photo Film Co., Tokyo, Japan).

Cell growth assay

Cells were seeded in a 12-well plate to 40% confluency for transfection. After transfection, cells were incubated with 0.5 mg/ml of MTT [3-(4,5-dimethylthiazol-2-yl)-2,5-diphenyltetrazolium bromide] solution (Biosesang, Seoul, Korea) for 1 hr. The dark blue formazan products formed by viable cells were dissolved in dimethyl sulfoxide (DMSO; Sigma, St. Louis, MO, USA), and absorbance was measured using a SYNERGY H1 Multilabel plate reader (Bio-Tek, Winooski, VT, USA).

5-Bromo-2'-deoxyuridine (BrdU) cell proliferation assay

Cells were seeded in a 24-well plate to 40% confluency for transfection. After transfection, BrdU reagent for 6h with cells. The assay was performed with a BrdU cell proliferation assay kit (Millipore) in accordance with the manufacturer's protocol every 24 hours. The final product was quantified at 450nm by SYNERGY H1 multilabel plate reader

Clonogenic assay

For clonogenic cell proliferation assay, negative control siRNA (si-Cont) and si-GAS5 transfected cells were seeded in 6-well plate (1000 cells/well). After 12days, colonies were fixed with 1% paraformaldehyde for 30 min at room temperature and stained with 0.5% crystal violet for

1 hour at room temperature. The stained colonies were counted using commercial software (Clono-counter).

Cell cycle analysis

Cells were transfected with siRNAs for each gene in 60 mm diameter cell culture dishes. After 48 hours incubation, cells were harvested by Trypsin-EDTA, washed with cold phosphate buffered saline (PBS), fixed in 70% ethanol, resuspended in 200 ul PBS containing 3 mg/ml RNase A (Sigma,), 50 ug/ml propidium iodide (PI, Sigma) and 1% Triton X-100. Cells incubated in the dark for 45 min at 37°C. Stained cells fractions were detected using FACS Canto^{TM} flow cytometer (BD Biosciences, San Jose, CA, USA).

Apoptosis analysis

The Annexin V-FITC Apoptosis Detection Kit I (BD Biosciences, San Jose, CA) was used to measure the level of apoptosis. After transfection or treatment for 48 hr, cells were harvested by Trypsin-EDTA, washed with PBS, and resuspended using 1 × binding buffer and 100 ul containing 1 × 105 cells were transferred to 5 ml culture tube. Then, 5 ul of Annexin V-FITC and 10 ul of propidium iodide (PI) solution were added. Cells were incubated in the dark for 15min at room temperature. After incubation, a 400 ul of 1 × binding buffer was put to each tube, and apoptotic fractions were detected using the FACS Canto^{TM} flow cytometer (BD Biosciences).

Boyden chamber motility and transwell invasion assays

For *in vitro* cell motility and invasion assays, cell motility was measured by modified Boyden

chamber assay. Cell invasion assay were used transwell plates and cell culture inserts (BD Biosciences). For invasion assay, Matrigel (BD Biosciences) was diluted to 0.3 mg/ml concentration with coating buffer (0.01 M Tris, 0.7% NaCl, pH 8.0) and 100μl Matrigel was coated onto the upper of the cell culture insert. After incubation for 1 hour at 37℃, the cell culture insert was ready for seeding. cells were seeded (1×10^5 cells/well for the motility assay, 1.5×10^5 cells/well for the invasion assay) into the cell culture insert with serum-free medium and lower transwell contained the presence of 2% FBS as chemoattractant. The plate was incubated for 12 hours at 37℃. After, stained for cells were using Diff-Quik staining kit (Sysmex, Kobe, Japan). Cells were photographed using an Axiovert 200 inverted microscope (Zeiss, Jena, Germany) at x200 magnification. Cells were counted in three random images.

Scratch wound healing assay

Cells were transfected for 24 hours in 60 mm diameter cell culture dishes. Then, cells were cells were harvested by Trypsin-EDTA, and 1×10^6 cells were seeded in wells of a 6-well cell culture plate. Incubation at overnight, scratch made on a uniform layer using a micropipette tip. Photographs of the same area of the wound were taken after 0 hour and 24 hours with IX70 photomicroscope (Olympus, Tokyo, Japan)

Random mutagenesis

Random mutagenesis was performed using a GeneMorph II Random Mutagenesis Kit (Agilent, CA, USA) in accordance with the manufacturer's protocol. Briefly, the total RNA was extracted from HCC cells and used as a template for PCR amplification with Mutazyme II DNA polymerase, provided in the mutagenesis kit, and GAS5-specific primers. The PCR products were purified using

a PCR purification kit (Cosmogenetech, Seoul, Korea) and subsequently cloned into the pcDNA3.1 expression vector. The mutated GAS5 sequence was confirmed by Sanger sequencing, and its secondary structure was predicted using the RNAfold web tool. GAS5 mutant sequence is
‘TTTCGAGGTAGGAGTCGACTCCTGTGAGGTATGGTGCTGGGTGCAGATGCAGTGTGGCTC
TGGATAGCACCTTATGGACAGTTGTGTCCCCAAGGAAGGATGAGAATAGCTACTGAAGTC
CTAAAGAGCAAGCCTAACTCAAGCCATTGGCACACAGGCATTAGACAGAAAGCTGGAAG
TTGAAATGGTGGAGTCCAACTTGCCTGGACCAGCTTAATGGTTCTGCTCCTGGTAACGTTT
TTATCCATGGATGACTTGCTTGGGTAAGGACATGAAGACAGTTCCTGTCATACCTTTTAAA
GGTATGGAGAGTCGGCTTGACTACACTGTGTGGAGCAAGTTTTAAAGAAGCAAAGGACTC
AGAATTCATGATTGAAGAAATGCAGGCAGACCTGTTATCCTAAACTAGGGTTTTTAATGAC
CACAACAAGCAAGCATGCAGCTTACTGCTTGAAAGGGTCTTGCCTCACCCAAGCTAGAGT
GCAGTGGCCTTTGAAGCTTACTACAACCTCGAACTTCTGGGCTCAAGTGATCCTCAGCCTC
CCAGTGGTCTTTGTAGACTGCCTGATGGAGTCTCATGGCACAAGAAGATTAAAACAGTGT
CTCCAATTTTAATAAATTTTTGCAATCC’.

Bioinformatics target analysis

To identify candidate genes regulated by m6A methylation at the subunit gene level, data from the TCGA_LIHC, ICGC_LIRI, and NCBI GEO databases, along with web tools (ENCORI, RMVAR), were utilized. A total of 39,136 genes predicted to have m6A methylation were identified through the RMVAR tool. The GSE90684 dataset, which contains gene expression data following shRNA-mediated knockdown of the IGF2BPs family (IGF2BP1, IGF2BP2, and IGF2BP3), was used to identify genes with reduced expression. Additionally, the GSE90639 dataset, based on RIP-seq results, provided gene expression data for genes that interact with the IGF2BPs family. Both GSE90684 and GSE90639 datasets were employed to select genes whose expression is influenced by m6A methylation and

regulated by the IGF2BPs family. Among the 148 genes selected, 51 genes, which showed consistent upregulation in both the TCGA_LIHC and GSE114564 datasets, were identified as genes whose expression is regulated by m6A methylation and the IGF2BPs family in the context of HCC. To identify microRNAs that interact with GAS5, the ENCORI web tool, which predicts microRNAs interacting with lncRNAs, was utilized. A total of 131 microRNAs were predicted by ENCORI to bind to GAS5. Experimentally, MS2-GAS5 was cloning, and MS2 binding protein was used for immunoprecipitation to identify microRNAs interacting with GAS5, which were confirmed by qPCR array (GeneCopoeia, MD, USA). We confirmed a common set of 20 microRNAs from both the predictions made by ENCORI and those experimentally validated. Additionally, 13 microRNAs with a TPM value greater than 1 in both the TCGA_LIHC and GSE114564 (Catholic_LIHC) datasets were selected. Finally, 5 microRNAs that showed consistent upregulation in the TCGA_LIHC, GSE114654, and GSE77276 (Tsinghua_LIHC) datasets were identified as the final candidate microRNAs.

Supplementary Table 1. Candidate RNAs with m6A methylations in HCC

No.	Gene	Gene type	GSE90684			TCGA_LIHC	Catholic_LIHC (GSE114564)	ICGC_LIRI	GSE77314
			shIGF2BP1	shIGF2BP2	shIGF2BP3				
1	UBE2C	protein coding	-2.50	-10.19	-1.98	6.11	12.55	11.13	26.05
2	CDC20	protein coding	-2.20	-8.98	-1.87	5.34	13.52	11.44	31.94
3	PTTG1	protein coding	-2.66	-2.41	-2.20	4.93	9.57	7.83	17.91
4	E2F1	protein coding	-1.98	-8.22	-1.96	4.87	10.12	8.66	15.71
5	CCNB1	protein coding	-2.92	-6.79	-2.21	4.80	8.22	8.75	16.57
6	TK1	protein coding	-3.98	-10.78	-4.54	4.70	4.86	4.88	6.15
7	MYBL2	protein coding	-3.27	-17.73	-3.03	4.64	21.27	11.50	37.41
8	TOP2A	protein coding	-2.33	-10.00	-3.62	4.52	16.62	11.97	20.35
9	RRM2	protein coding	-1.96	-11.89	-3.64	3.95	10.33	7.80	12.59
10	GAS5	lincRNA	-2.38	-3.59	-1.88	3.90	1.63	2.63	2.76
11	ZWINT	protein coding	-2.10	-7.50	-2.46	3.76	5.60	6.03	8.65
12	MCM2	protein coding	-2.51	-2.48	-2.17	3.55	4.59	5.21	8.85
13	MCM3	protein coding	-2.58	-2.93	-2.29	3.39	2.31	3.38	5.01
14	FEN1	protein coding	-1.88	-4.15	-3.58	3.29	2.94	3.69	5.98
15	KPNA2	protein coding	-1.51	-1.94	-2.10	3.28	2.60	3.73	7.77

16	CDK1	protein coding	-2.59	-22.36	-2.91	3.28	9.06	8.81	14.78
17	RECQL4	protein coding	-2.10	-2.59	-1.64	3.22	3.24	4.23	10.52
18	IRAK1	protein coding	-1.73	-2.06	-1.54	3.20	1.78	3.26	3.87
19	CDT1	protein coding	-2.69	-8.75	-1.91	3.17	3.48	7.37	9.01
20	MCM7	protein coding	-2.56	-4.47	-2.90	3.16	1.93	2.80	2.85
21	AURKB	protein coding	-1.82	-11.03	-1.74	3.09	5.41	7.89	12.46
22	CDCA5	protein coding	-2.52	-12.29	-2.10	3.03	8.22	9.42	15.54
23	H2AFZ	protein coding	-1.64	-2.34	-1.93	2.94	1.55	2.71	3.66
24	MCM4	protein coding	-1.86	-3.67	-3.13	2.84	2.41	3.97	4.59
25	TACC3	protein coding	-1.77	-5.56	-1.50	2.77	2.70	3.51	4.86
26	MCM5	protein coding	-3.40	-5.57	-2.71	2.77	1.75	2.73	4.41
27	CDC6	protein coding	-2.34	-6.80	-2.45	2.63	10.29	6.29	9.13
28	TROAP	protein coding	-2.42	-14.84	-1.70	2.60	10.92	10.33	21.35
29	MKI67	protein coding	-2.32	-15.20	-1.91	2.58	9.53	8.36	15.47
30	GLA	protein coding	-1.76	-1.58	-1.58	2.57	1.69	2.45	3.05
31	PLK1	protein coding	-2.20	-7.53	-1.95	2.57	5.20	6.40	5.67
32	CKAP4	protein coding	-1.56	-1.66	-3.09	2.52	2.00	2.83	2.85
33	TUBA1B	protein coding	-2.21	-3.25	-2.01	2.43	1.51	2.95	4.08

34	NCAPG	protein coding	-2.03	-8.93	-2.45	2.41	16.49	4.51	11.39
35	LMNB1	protein coding	-1.73	-7.71	-1.75	2.31	2.16	2.72	4.64
36	PAQR4	protein coding	-1.76	-1.71	-3.10	2.13	2.84	3.86	5.41
37	CSE1L	protein coding	-1.51	-2.21	-2.52	2.12	1.60	2.06	2.21
38	C16orf59	protein coding	-1.94	-2.03	-1.58	2.10	6.59	5.42	6.57
39	KIF18B	protein coding	-2.14	-15.41	-1.66	2.02	19.41	7.77	24.36
40	TTLL4	protein coding	-1.80	-1.72	-1.77	1.97	2.27	2.52	2.51
41	NDOR1	protein coding	-1.72	-1.77	-1.54	1.96	1.61	2.03	1.49
42	CHTF18	protein coding	-2.54	-2.42	-1.59	1.93	1.70	2.49	2.53
43	PKMYT1	protein coding	-3.01	-3.76	-2.21	1.90	5.91	4.56	5.76
44	HSPH1	protein coding	-1.58	-1.91	-1.96	1.90	1.54	1.26	1.72
45	COCH	protein coding	-1.90	-1.62	-2.10	1.88	6.86	12.96	13.62
46	MAD2L1	protein coding	-2.04	-9.79	-1.85	1.88	4.00	5.34	6.31
47	CDC23	protein coding	-1.70	-1.70	-1.54	1.82	1.62	2.09	1.44
48	RFC5	protein coding	-1.69	-1.80	-1.90	1.72	1.52	1.95	1.58
49	CBX2	protein coding	-7.65	-4.63	-2.96	1.62	4.22	5.64	6.23
50	CDCA7	protein coding	-2.60	-3.61	-3.73	1.60	9.12	7.39	19.82
51	KNOP1	protein coding	-1.77	-2.18	-2.06	1.51	1.68	1.95	1.47

Supplementary Table 2. GAS5 fold change in GENT and TCGA

No.	Organ	GENT F.C.	TCGA project	TCGA F.C.
1	Liver	2.10	LIHC	3.17
2	Colon	2.06	COAD	2.12
			KICH	-1.18
3	Kidney	1.89	KIRC	2.97
			KIRP	1.65
4	Bladder	1.77	BLCA	1.32
5	Brain	1.70	GBM	2.10
6	Prostate	1.55	PRAD	1.91
7	Stomach	1.31	STAD	1.58
8	Lung	1.27	LUAD	1.96
			LUSC	1.74
9	Thyroid	1.27	THCA	1.62
10	Skin	1.15	SKCM	-1.37
11	Ovary	1.09	OV	N/A
12	Endometruim	1.03	UCEC	-1.07

N/A: Not available

F.C.: Fold Change

Supplementary Table 3. Pearson's positive correlation between GAS5 and ceRNA candidates

No.	Genes	TCGA_LIHC	Catholic_LIHC	ICGC_LIRI	GSE77314	Mean of Pearson's correlation in HCC
1	SMARCA4	0.561	0.241	0.357	0.762	0.480
2	JRK	0.582	0.277	0.455	0.410	0.431
3	ZSCAN2	0.591	0.314	0.252	0.539	0.424
4	LPCAT1	0.452	N/A	0.331	0.484	0.422
5	CCDC137	0.617	0.073	0.390	0.425	0.376
6	PABPC1	0.000	0.619	0.371	0.508	0.375
7	RNF220	0.562	0.326	0.401	0.180	0.367
8	ADPRHL2	0.417	0.348	0.304	0.245	0.329

Supplementary Table 4. List of antibodies used in western blot analysis and Chromatin immunoprecipitation

protein	Manufacturer	Catlog No.	Application	Dilution
AGO2	MBL life science	RN003M	Western blot	1:1000
caspase3	Cell signaling	#9668	Western blot	1:1000
cleaved caspase3	Cell signaling	#9661	Western blot	1:500
CDK2	Cell signaling	#2546	Western blot	1:1000
CDK4	Cell signaling	#12790	Western blot	1:1000
CDK6	Cell signaling	#3136	Western blot	1:1000
cleaved PARP	Cell signaling	#9541	Western blot	1:1000
Cyclin D1	Cell signaling	#2978	Western blot	1:1000
Cyclin E	Santa Cruz	sc-481	Western blot	1:1000
E-cadherin	BD Transduction	610404	Western blot	1:1000
Fibronectin	Santa Cruz	sc-9068	Western blot	1:1000
GAPDH	Santa Cruz	sc-32233	Western blot	1:1000
IGF2BP1	Cell signaling	#8482	Western blot	1:1000
IGF2BP2	Cell signaling	#14672	Western blot	1:1000
IGF2BP3	Cell signaling	#57145	Western blot	1:1000
m6A	Synaptic Systems	202 003	Western blot	1:1000
METTL3	abcam	195352	Western blot	1:1000
N-cadherin	BD Transduction	610920	Western blot	1:1000
p27	Santa Cruz	#3686	Western blot	1:1000
PARP	Cell signaling	#9542	Western blot	1:1000
p-pRb	Cell signaling	#9308	Western blot	1:1000
Slug	Assay Biotech	R12-2357	Western blot	1:1000
SMARCA4	Santa Cruz	sc-10768	Western blot	1:1000
Smarca4	BETHYL	A303-877A	Western blot	1:1000
Snail	Assay Biotech	B1235	Western blot	1:1000
Vimentin	GeneTex	GTX100619	Western blot	1:1000
AGO2	MBL life science	RN003M	Immunoprecipitation	1:50
IGF2BP1	Cell signaling	#8482	Immunoprecipitation	1:50
IGF2BP2	Cell signaling	#14672	Immunoprecipitation	1:50
IGF2BP3	Cell signaling	#57145	Immunoprecipitation	1:50

m6A	Synaptic Systems	202 003	Immunoprecipitation	1:50
METTL3	abcam	195352	Immunoprecipitation	1:50

Supplementary Table 5. List of primer sequences used in PCR experiments

Gene	Primer	Nucleotide sequence	experiments
GAS5	Forward	5'- AGCTGGAAGTTGAAATGG -3'	qRT-PCR
	Reverse	5'- CAAGCCGACTCTCCATACC -3'	
	Forward	5'- TTTCGAGGTAGGAGTCGACT -3'	RT-PCR
	Reverse	5'- TTAATAAATTTTTGCAATCC -3'	
SMARCA4	Forward	5'- CATCATCGTGCCTCTCTCAA -3'	qRT-PCR
	Reverse	5'- TTCAACGTCTTGCTGACGAC -3'	
Gas5	Forward	5'- TCTCACAGCCAGTTCTGTGG -3'	qRT-PCR
	Reverse	5'- TTGCAGTGCCTTCACTTGAG -3'	
mmu-miR-423-3p	Forward	5'- AGCUCGGUCUGAGGCCCCUCAGU -3'	qRT-PCR
	Reverse	5'- GTGCAGGGTCCGAGGT -3'	
U6	Forward	5'- GTGCTCGCTTCGGCAGCA -3'	qRT-PCR
	Reverse	5'- CAAATATGGAACGCTTC -3'	
GAPDH	Forward	5'- ACCAGGTGGTCTCCTCTGAC -3'	qRT-PCR
	Reverse	5'- TGCTGTAGCCAAATTCGTTG -3'	
Gapdh	Forward	5'- CCACCCAGAAGACTGTGGAT -3'	qRT-PCR
	Reverse	5'- CACATTGGGGGTAGGAACAC -3'	

Supplementary Figure & Figure legends

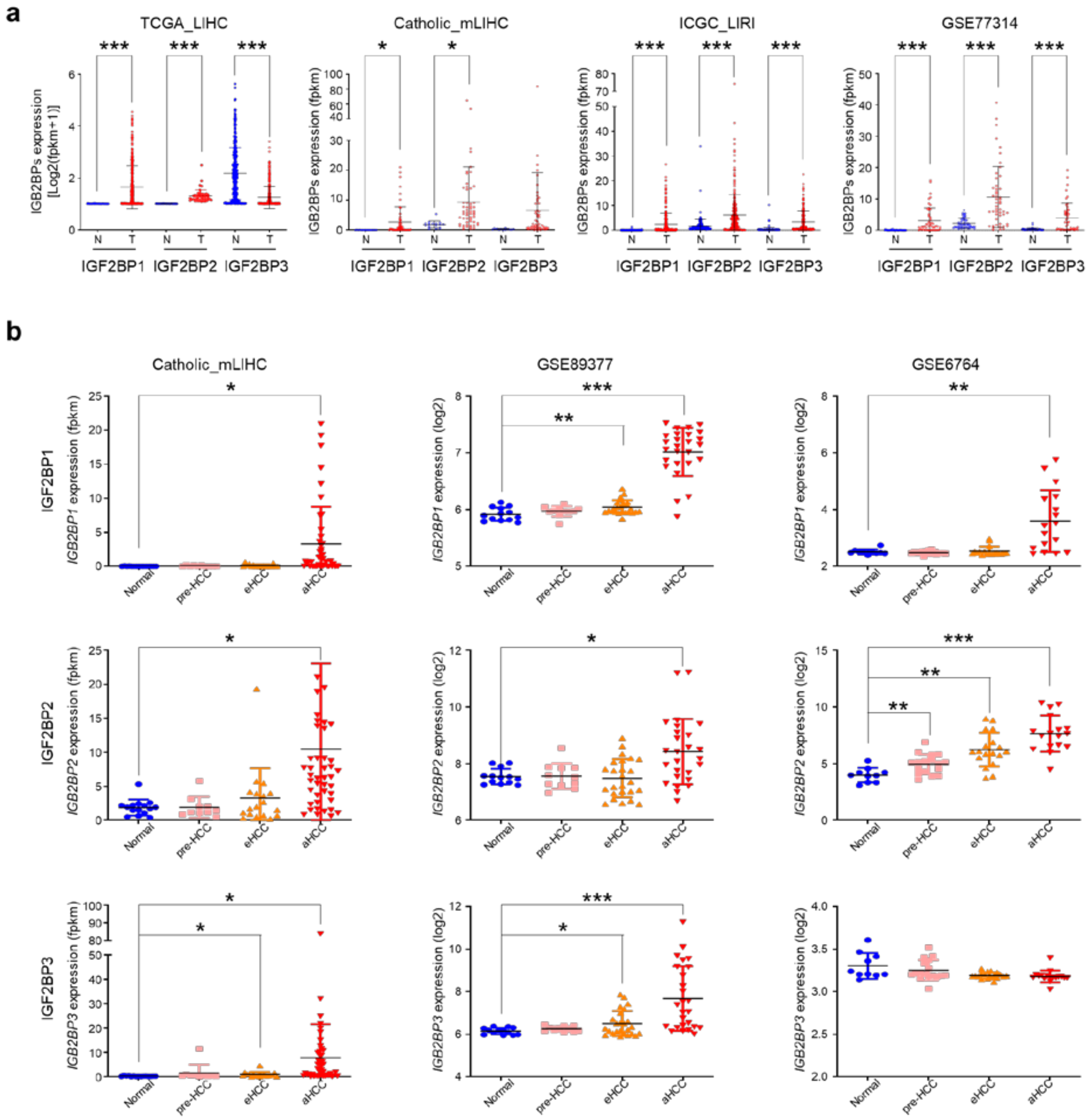

Supplementary Fig. 1 Overexpression of IGF2BP members positively correlated with advanced HCC. a Expression-pattern of IGF2BP family members was compared between HCC patients and healthy normal with TCGA_LIHC, Catholic_mLIHC (GSE114564), ICGC_LIRI, and GSE77314 datasets. **b** Expression-pattern of IGF2BP1 (top), IGF2BP2 (middle), and IGF2BP3 (bottom) were compared among multistage HCC patients of Catholic_mLIHC, GSE89377, and GSE6764 datasets. aHCC advanced hepatocellular carcinoma, eHCC early hepatocellular carcinoma, mLIHC multi-stage liver hepatocellular carcinoma.

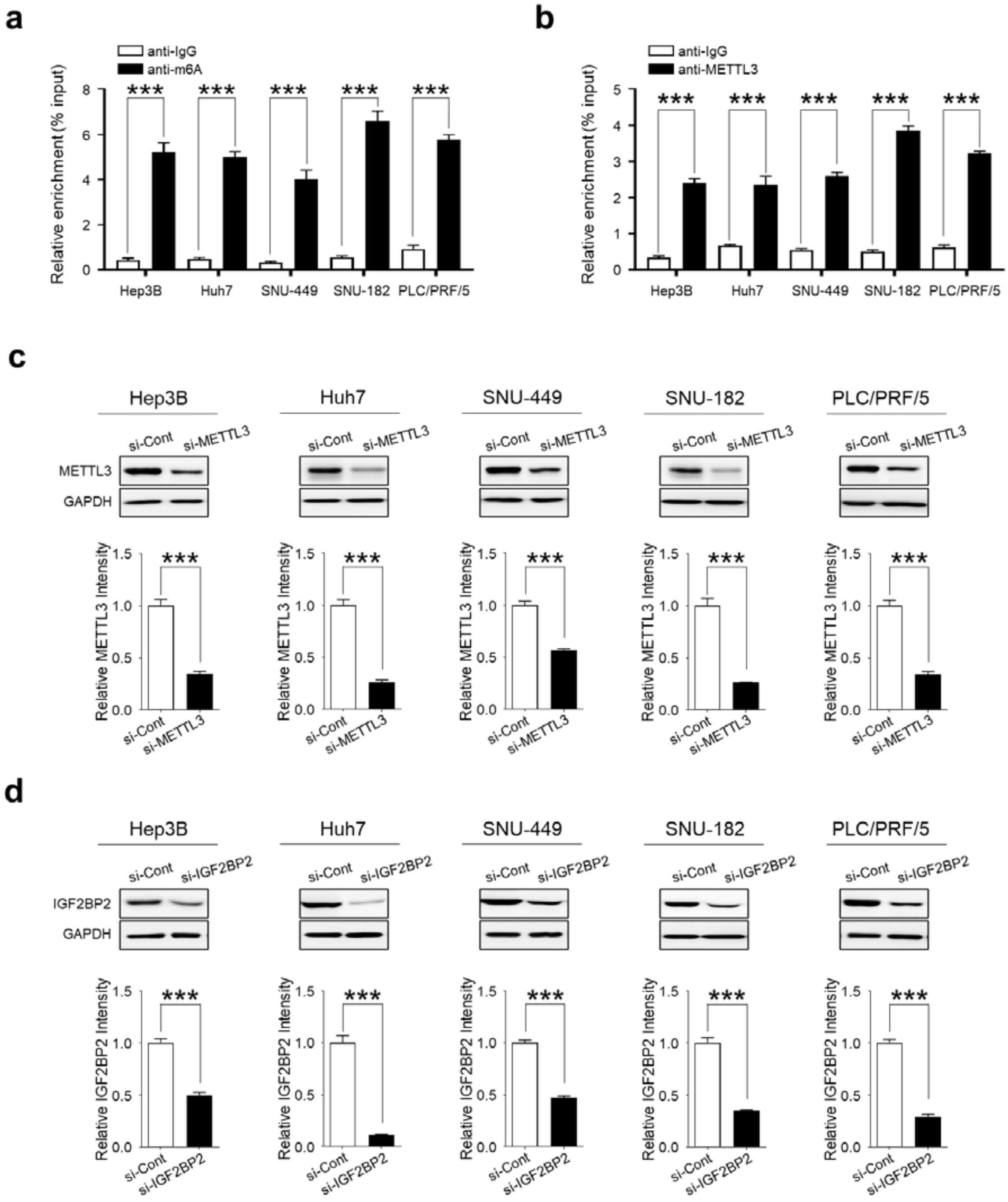

Supplementary Fig. 2 GAS5 (or m6A modified GAS5) expression was regulated by m6A- or METTL3 (writer) and IGF2BP2 (reader). **a, b** The RNA-IP (RIP) assays were performed to assess m6A methylated GAS5, using control IgG, anti-m6A **(a)**, or anti-METTL3 **(b)** in Hep3B, Huh7, SNU-449, SNU-182, or PLC/PRF/5 HCC cell lines. The amount of precipitated RNA by indicated antibodies is shown as relative fold enrichment. **c, d** Reduced protein detected by western

blot and densitometric analysis in HCC cell lines, after transfected with si-METTL3 **(c)** or si-IGF2BP2 **(d)**. RIP RNA immunoprecipitation.

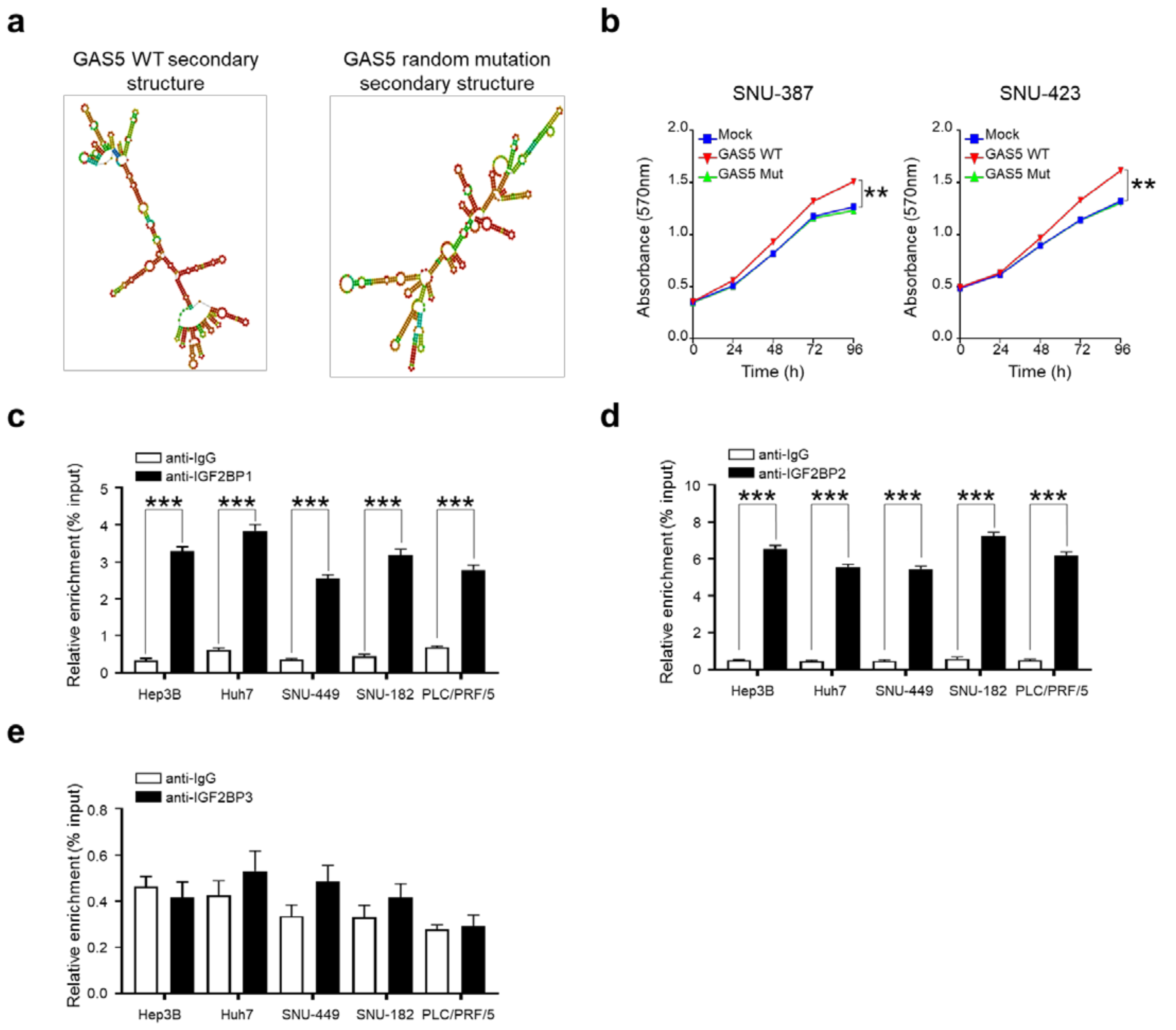

Supplementary Fig. 3 GAS5 (or m6A modified GAS5) expression was enriched by IGF2BP2 (reader)-bounded RNA immunoprecipitations. a RNAfold web server designed secondary structures of GAS5 WT and GAS5 random mutations. **b**. The growth of SNU-387 and SNU-423 HCC cells was determined by MTT assay after being transfected with GAS5 WT or GAS5 mutant (Mut) vectors. **c-e** The RNA-IP assays were performed using control IgG, anti-IGF2BP1 **(c)**, anti-IGF2BP2 **(d)**, or anti-IGF2BP3 **(e)** in Hep3B, Huh7, SNU-449, SNU-182, or PLC/PRF/5 HCC cell lines. The amount of precipitated

RNA by indicated antibodies is shown as relative fold enrichment. WT wild-type, Mut Mutation type.

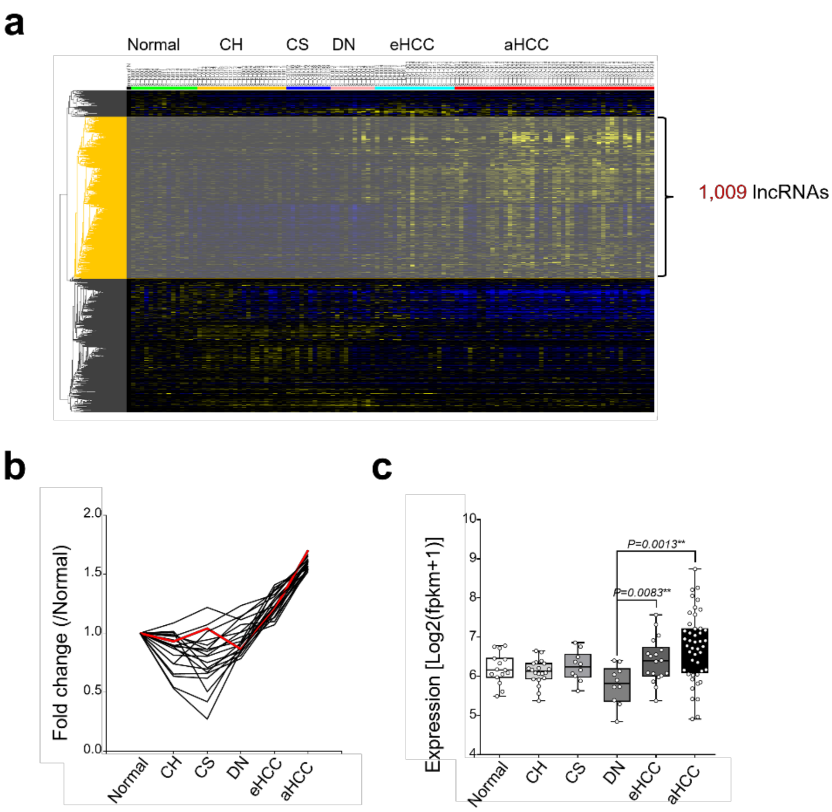

Supplementary Fig. 4 GAS5 is associated with advanced HCC. a Heatmap of 1,009 lncRNAs expression were significantly upregulated in advanced HCC, when analyzed multistage HCC patients of Catholic_mLIHC (GSE114564). **b** Driver lncRNAs expression-pattern with fold-change was analyzed in multistage HCC patients of Catholic_mLIHC (GSE114564). Red curve line indicates GAS5. **c** Expression of GAS5 (a driver lncRNA) was analyzed in multistage HCC patients of Catholic_mLIHC. All data are shown as the mean ± SEM, $^{**}p < 0.01$; unpaired t test. CH chronic hepatitis, CS cirrhosis, DN Dysplastic nodules, eHCC early HCC, aHCC advanced HCC.

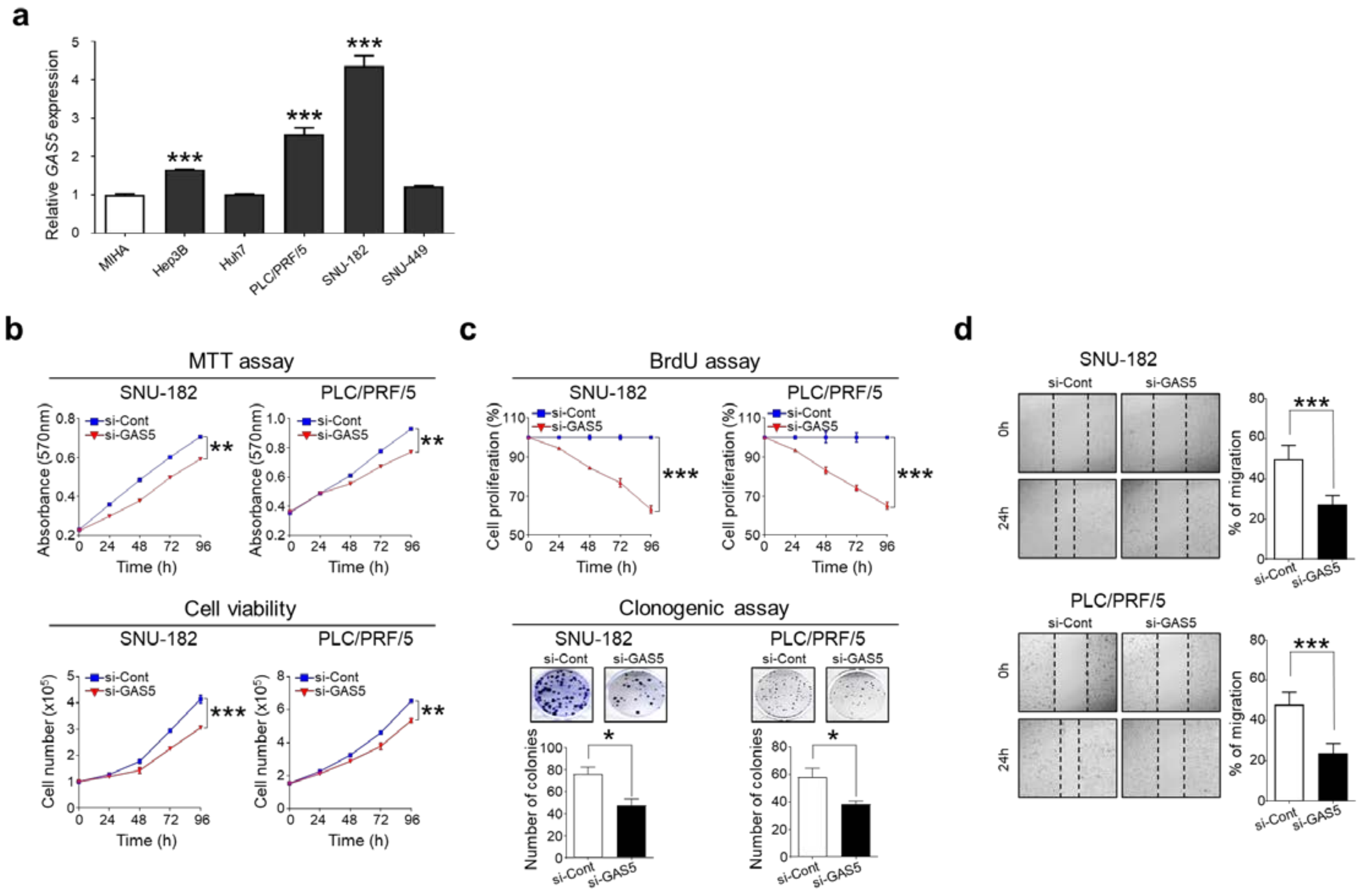

Supplementary Fig. 5 Inhibition of GAS5 expression regulated cell growth, proliferation and migration in GAS5-overexpressed HCC cell lines. a Endogenous RNA expression of GAS5 in HCC cell lines (Hep3B, Huh7, PLC/PRF/5, SNU-182, and SNU-449) including normal cell line (MIHA) was analyzed by qRT-PCR. **b** The cell growth (top) or cell viability (bottom) was determined by MTT assay in highly GAS5-overexpressed HCC cell lines (SNU-182 and PLC/PRF/5), after transfected with si-Cont or si-GAS5. **c** The BrdU assay (top), and clonogenic assay (bottom) were performed to measure cellular growth after transfected with si-Cont or si-GAS5 in SNU-182 and PLC/PRF/5. **d** Scratch wound healing assay was evaluated by migrated cell images (left) and a bar graph (right) with the percentage of migration, after transfected with si-Cont or si-GAS5 in SNU-182 and PLC/PRF/5. All data are shown as the mean ± SEM, *p < 0.05, **p < 0.01, ***p < 0.001; unpaired t test.

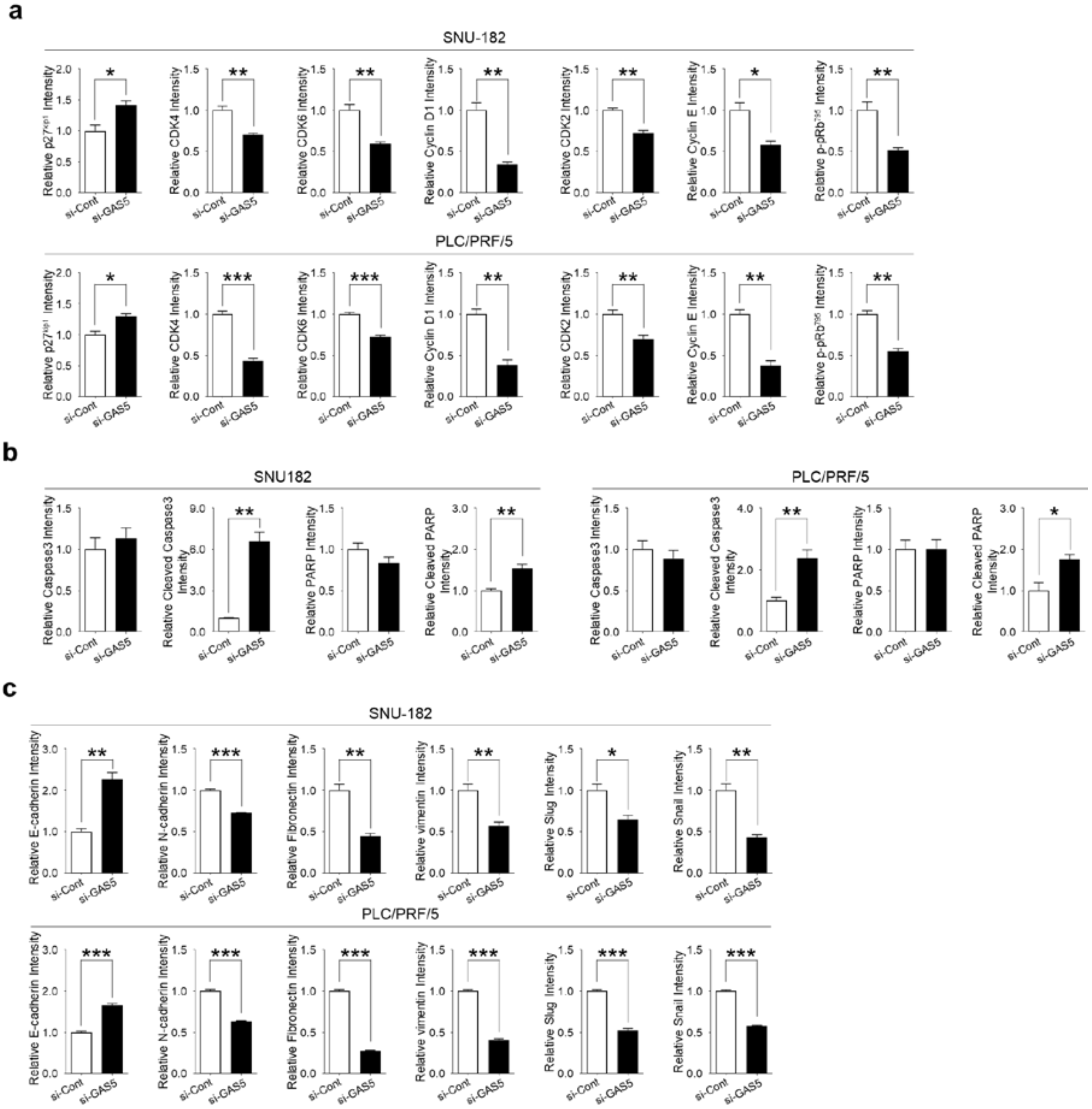

Supplementary Fig. 6 Densitometric analysis was performed to measure the protein intensity of the modulator in HCC cells. The expression of modulators proteins of Cell cycle **(a)**, Apoptosis **(b)**, and EMT **(c)** were quantified on a western blot using imageJ software.

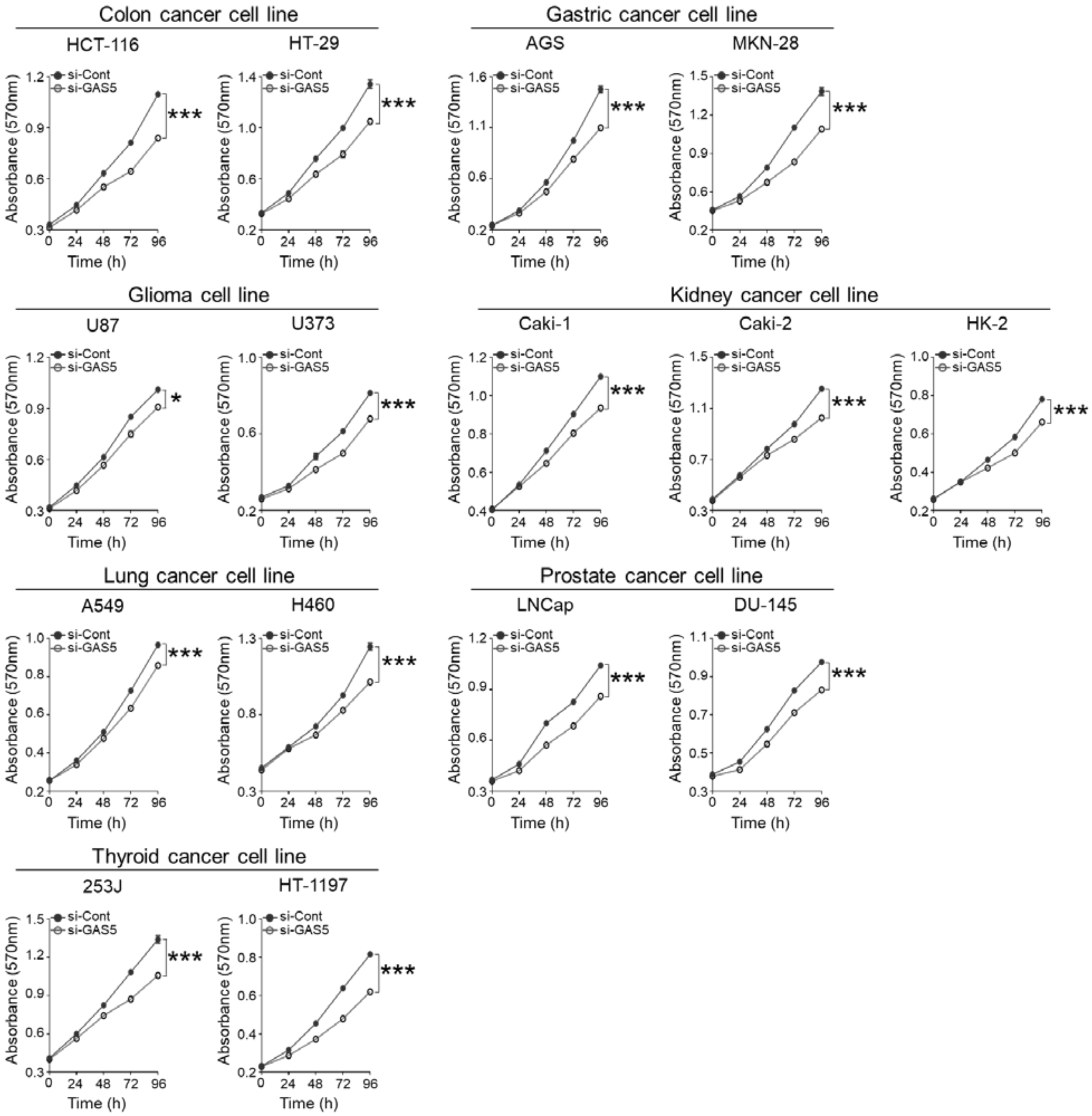

Supplementary Fig. 7 *In vitro* tumor cell growth of GAS5 in cancer cells. cell growth of each cancer cells transfected with si-Cont and si-GAS5 was determined by MTT assay.

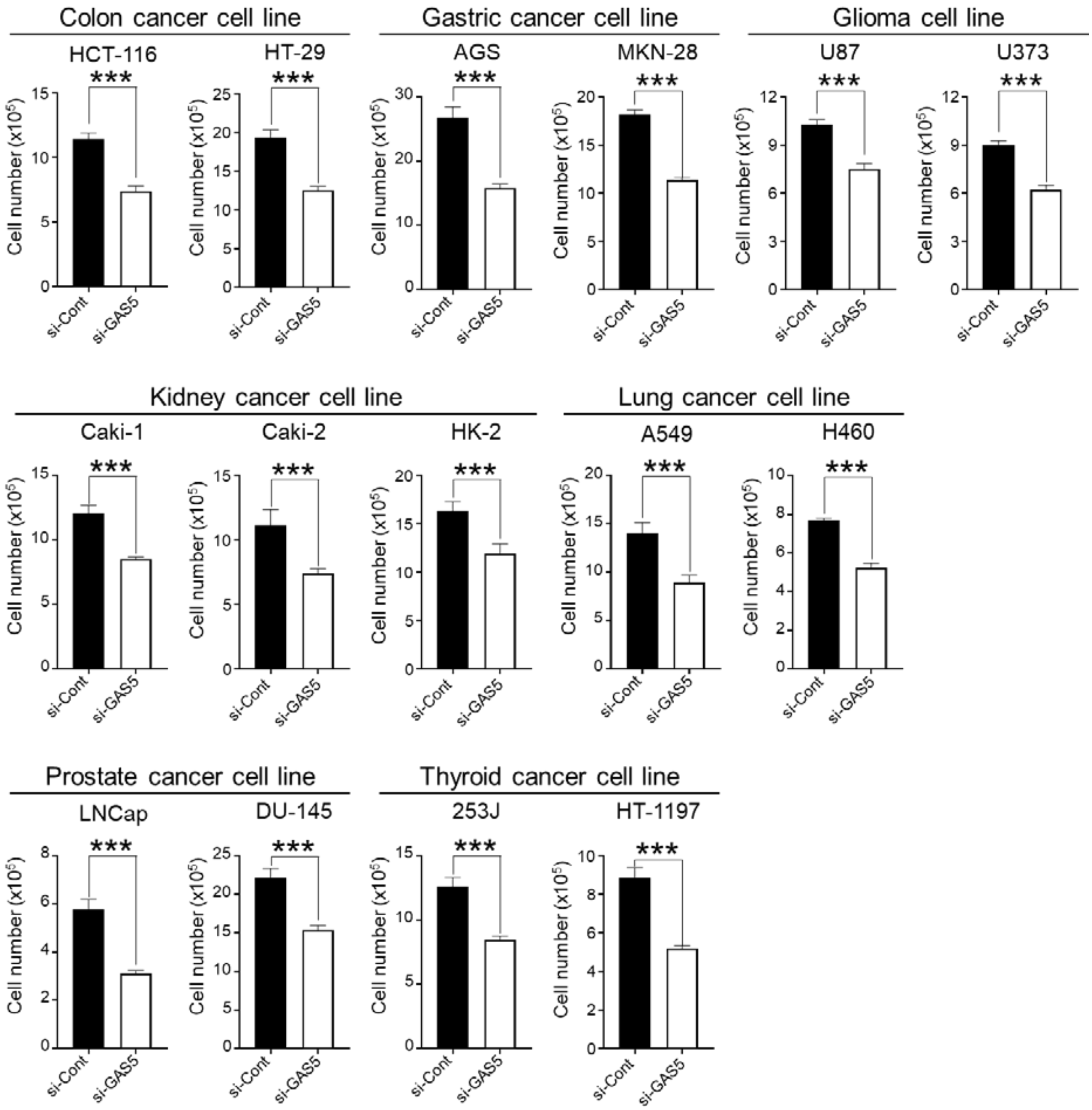

Supplementary Fig. 8 *In vitro* tumor cell viability of GAS5 in cancer cells. cell viability of each cancer cells transfected with si-Cont and si-GAS5 was determined by cells were counted using hemocytometer.

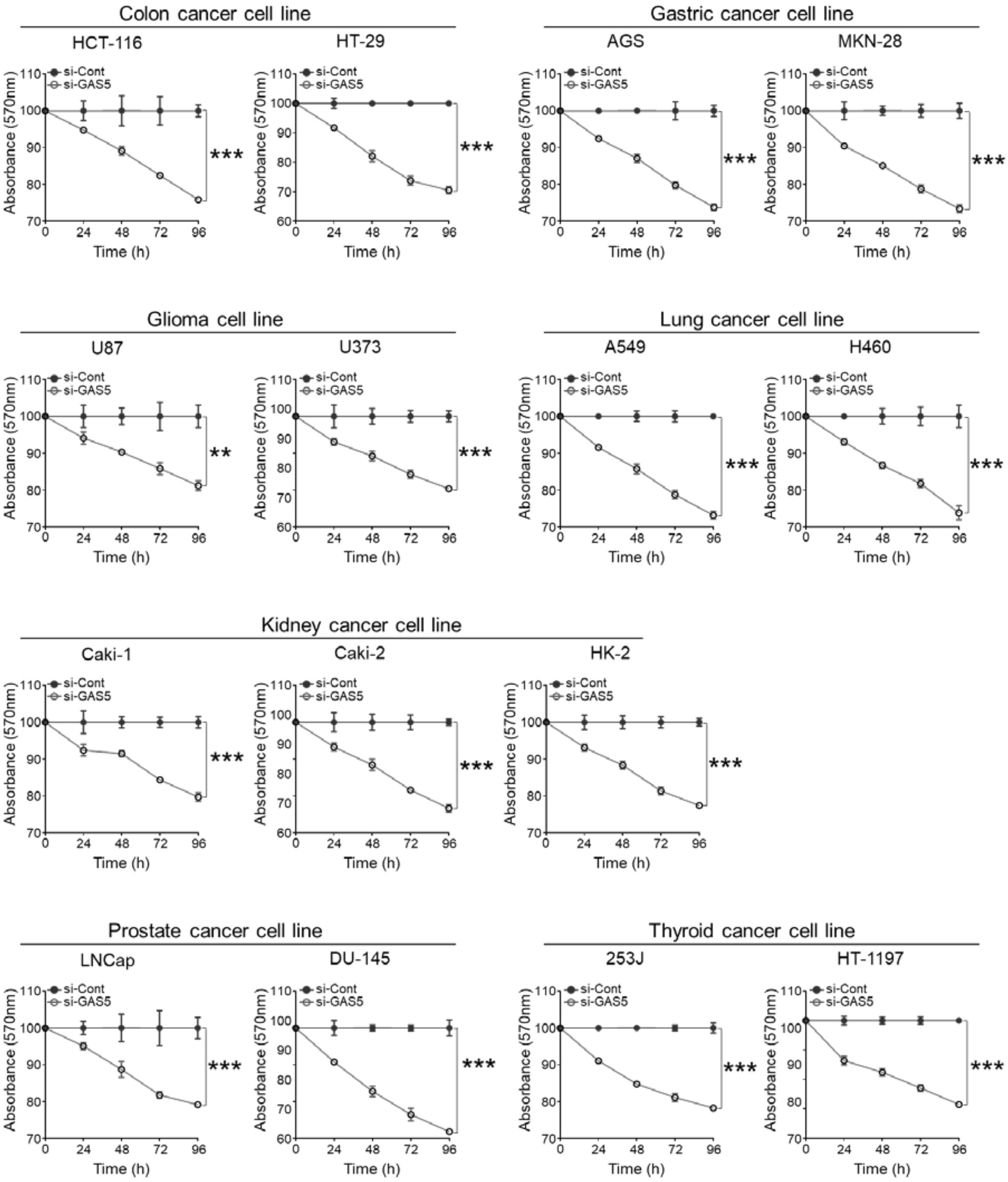

Supplementary Fig. 9 *In vitro* tumor cell proliferation of GAS5 in cancer cells. cell proliferation of each cancer cells transfected with si-Cont and si-GAS5 was determined by BrdU assay.

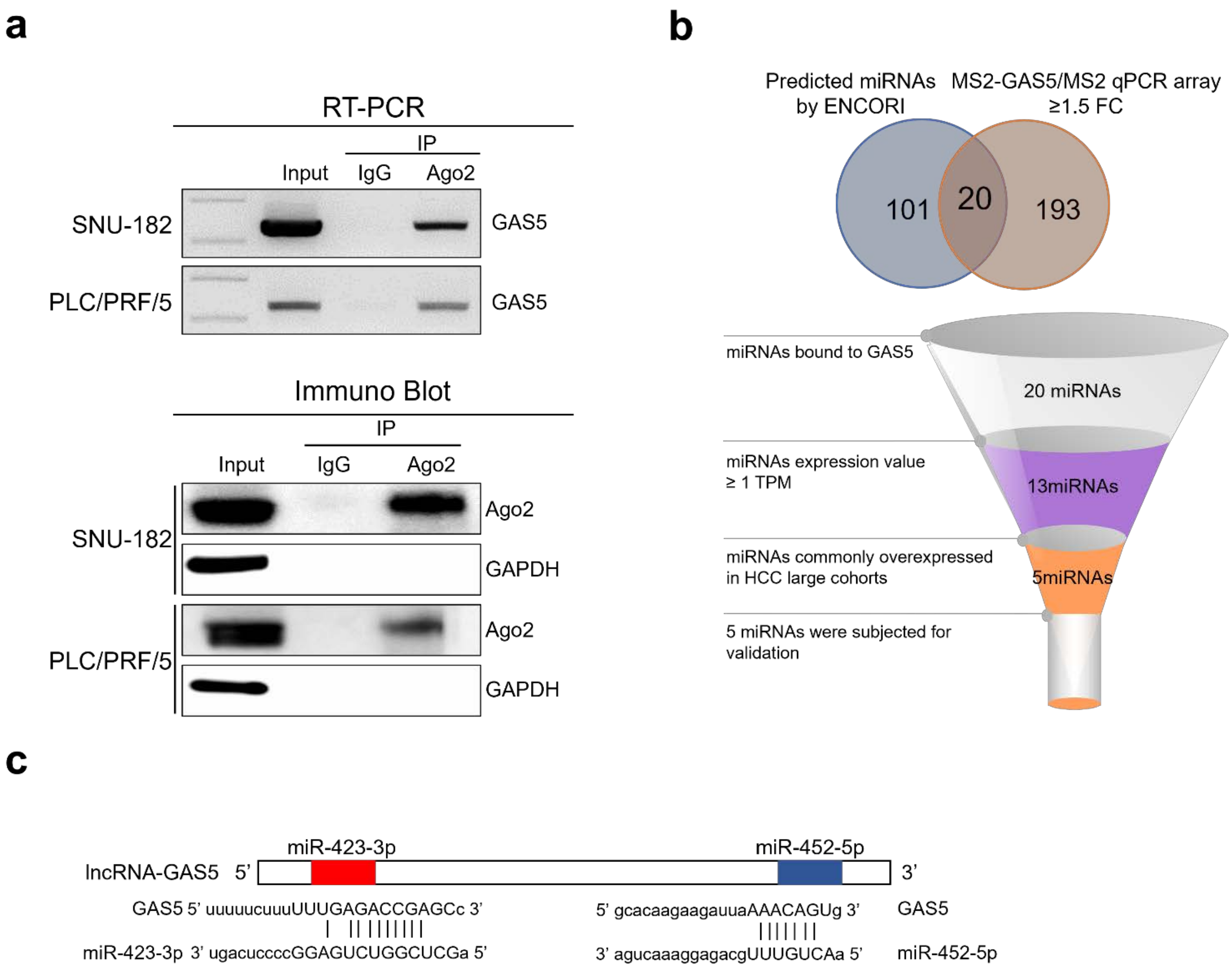

Supplementary Fig. 10 Identification of miRNAs binding to GAS5 through comprehensive analysis. a Ago2-IP assay was performed to evaluate GAS5 binding on miRNAs. GAS5 binding to miRNAs was verified by RT-PCR and western blot analysis in SNU-182, or PLC/PRF/5 HCC cell lines. **b** Venn diagram shows overlapped miRNAs of GAS5 binding miRNAs predicted ENCORI and qPCR array (top). The schematic strategy image shows how to identify GAS5 binding miRNAs (bottom). **c** Schematic presentation indicates the miR-423-3p (red) and miR-452-5p (blue) targeting sites in GAS5. IP Immunoprecipitation, TPM Transcripts per million.

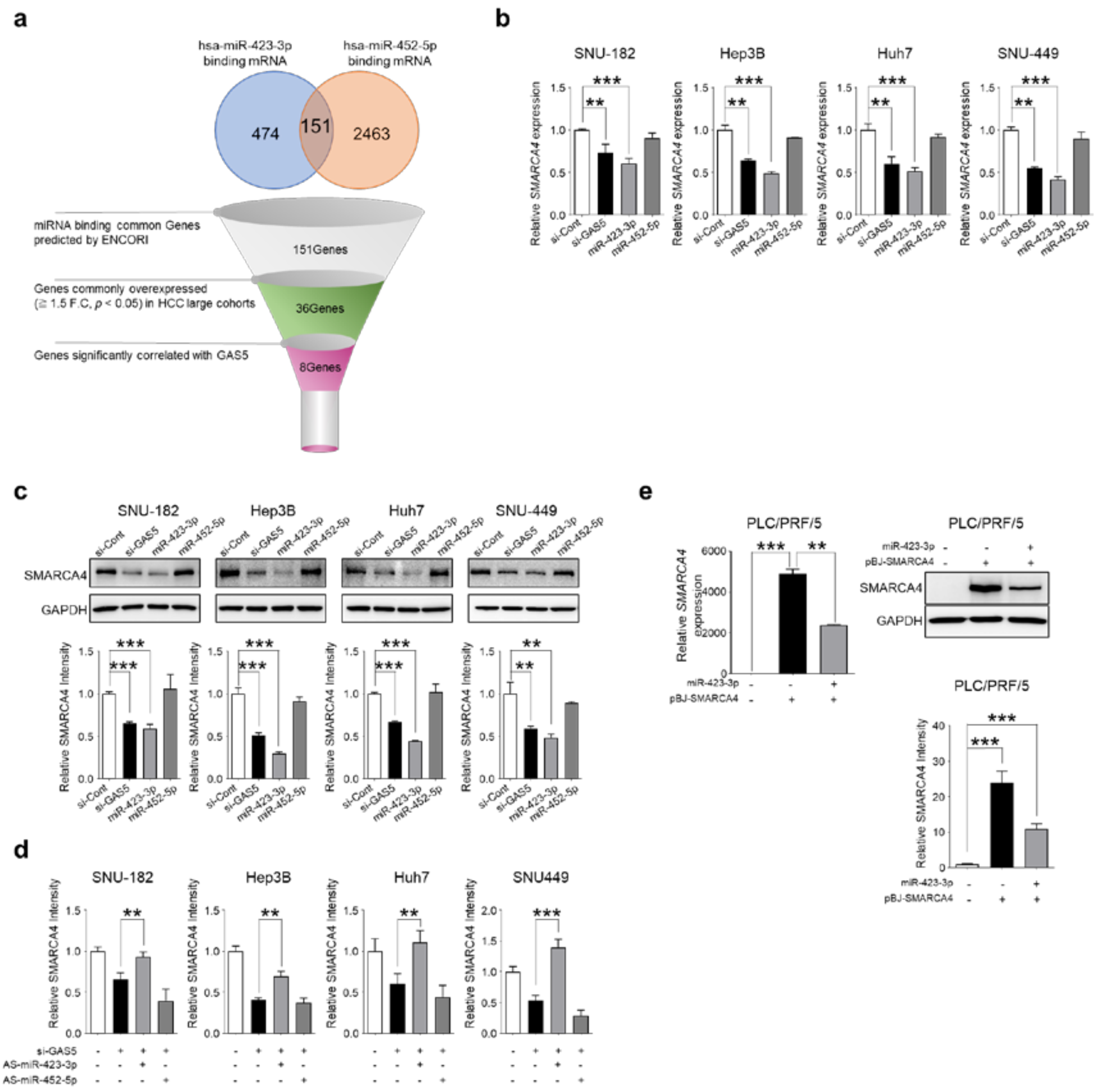

Supplementary Fig. 11 The miR-423-3p was identified as specifically binding miRNA to both GAS5 and SMARCA4 mRNA in HCC cell lines. a Venn diagram shows that 151 mRNAs were identified using overlapped genes of miRNA targets, miR-423-3p and miR-452-5p (top). Schematic strategy image how eight ceRNAs including SMARCA4 with similar GAS5 expression correlations, were identified in HCC database (bottom). **b, c** Relative expression of SMARCA4 was measured with qRT-PCR analysis **(b)** or western blotting analysis **(c)** in HCC cells, after transfected with si-Cont, si-GAS5, miR-423-3p, miR-452-5p mimics. **d** Densitometric analysis of SMARCA4 protein expression intensity following transfection with si-GAS5, followed by rescue of SMARCA4 with AS-miR-423-3p or AS-miR-452-5p. **e** The SMARCA4 null HCC cell line, PLC/PRF/5 was co-transfected with pBJ-SMARCA4, and miR-423-3p mimic sequentially. SMARCA4

expression was then determined using qRT-PCR and western blotting analysis. All data are shown as the mean ± SEM, $**p < 0.01$, $***p < 0.001$; unpaired t test.

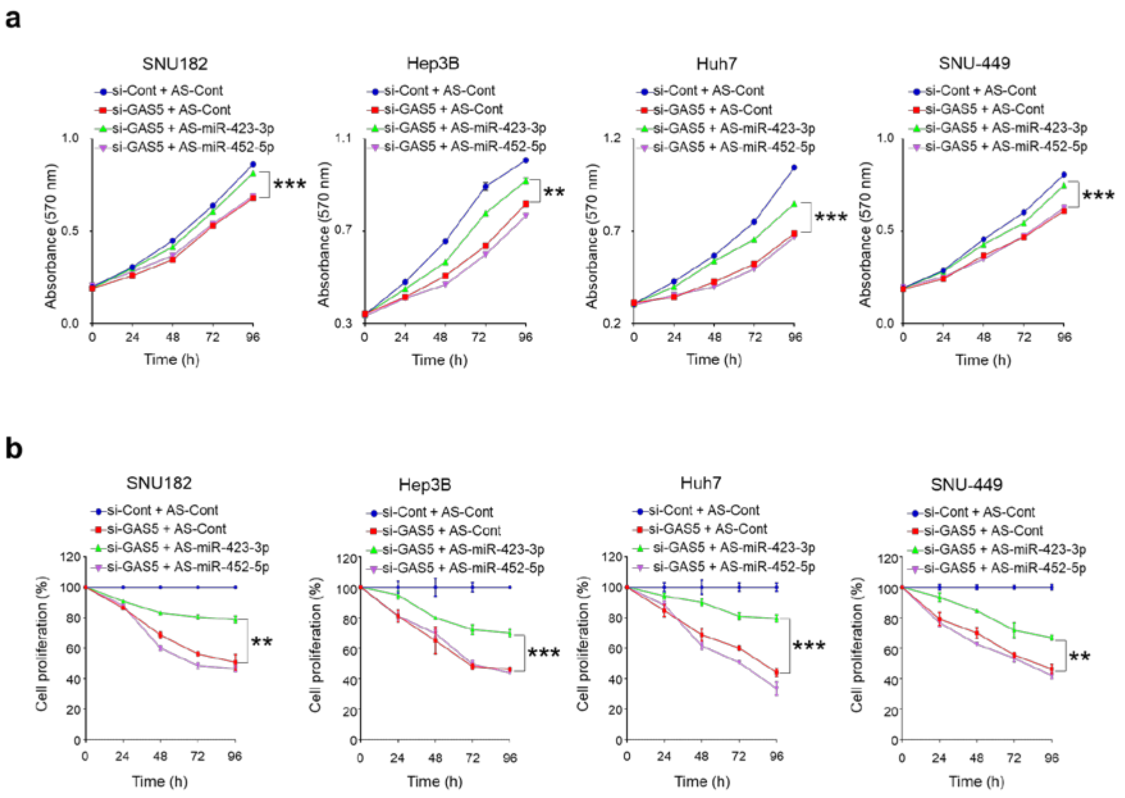

Supplementary Fig. 12 The tumorigenic potential of GAS5 mediated by SMARCA4 reactivation following miR-423-3p inhibition. The cell growth was measured by MTT assay **(a)** and cell proliferation by BrdU assay **(b)** in HCC cells (SNU182, Hep3B, Huh7, and SNU-449). All data are shown as the mean ± SEM, $**p < 0.01$, $***p < 0.001$; unpaired t test.

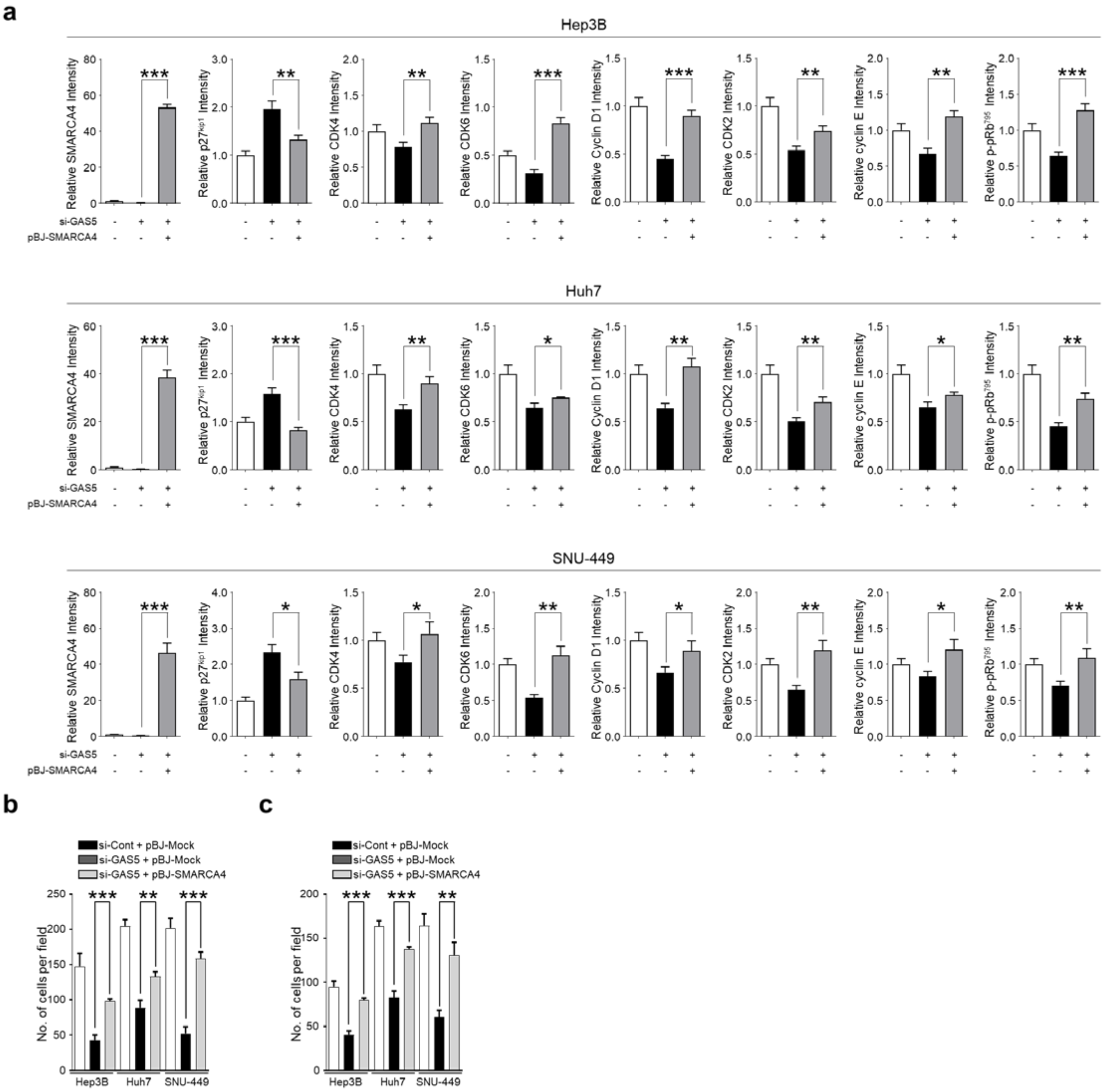

Supplementary Fig. 13 Rescue of SMARCA4 expression led to the recovery of tumorigenic potential. a Densitometric analysis of cell cycle modulators protein expression in Hep3B, Huh7, and SNU-449 HCC cells following si-GAS5 knockdown and subsequent rescue of SMARCA4 through pBJ-SMARCA4 transfection. **b, c** The bar graph represents the

number of migrated Hep3B, Huh7, and SNU-449 HCC cells. All data are shown as the mean ± SEM, *$p < 0.05$, **$p < 0.01$, ***$p < 0.001$; unpaired *t* test.

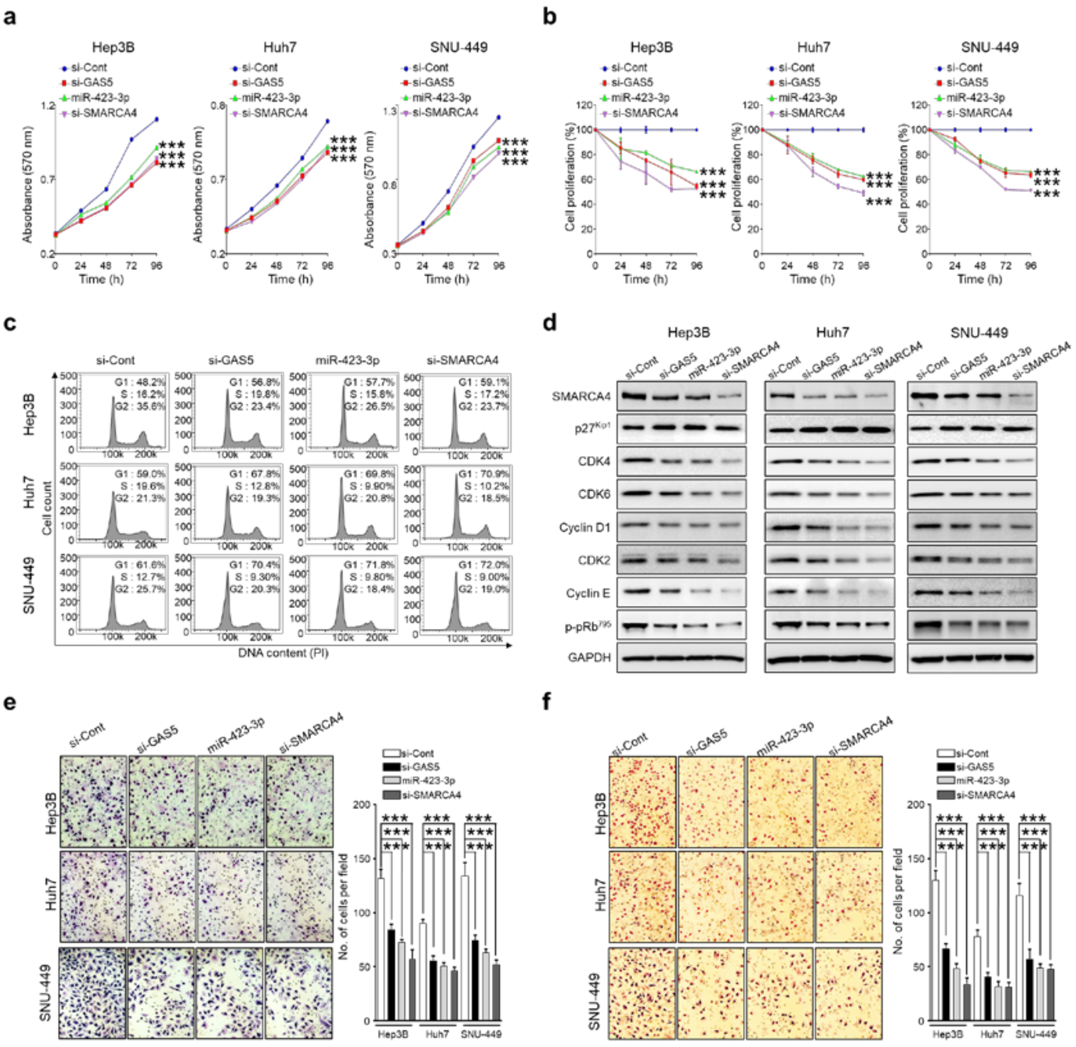

Supplementary Fig. 14 Knocking-out of either GAS5 or SMARCA4 expression inhibits tumorigenesis with similar phenotypes of overexpressed miR-423-3p in Hep3B, Huh7 and SNU-449 HCC cell lines. a-b The cell growth was measured by MTT assay **(a)** or BrdU assay **(b)**, after transfections of si-Cont, si-GAS5, si-SMARCA4, and miR-423-3p mimic in HCC cells (Hep3B, Huh7 and SNU-449). **c** Cell cycle profiles were analyzed after transfections of si-Cont, si-GAS5, si-SMARCA4, and miR-423-3p mimic in HCC cells. The bar graph indicates the percentage at each cell cycle phase in HCC cells. **d** Cell cycle modulators were analyzed with western-blot analysis in HCC cells after transfected with si-Cont or siRNA specific for indicated gene, and miR-423-3p mimic in HCC cells. GAPDH was used as a loading control. **e-f** Boyden chamber motility assay **(e)** and transwell invasion assay **(f)** were performed with analysis of cell images in HCC cells after transfections of si-Cont, si-GAS5, si-SMARCA4, and miR-423-3p mimic in HCC cells. The bar graph shows number of migrated HCC cells. All data are shown as the mean ± SEM, ***p < 0.001; unpaired t test.

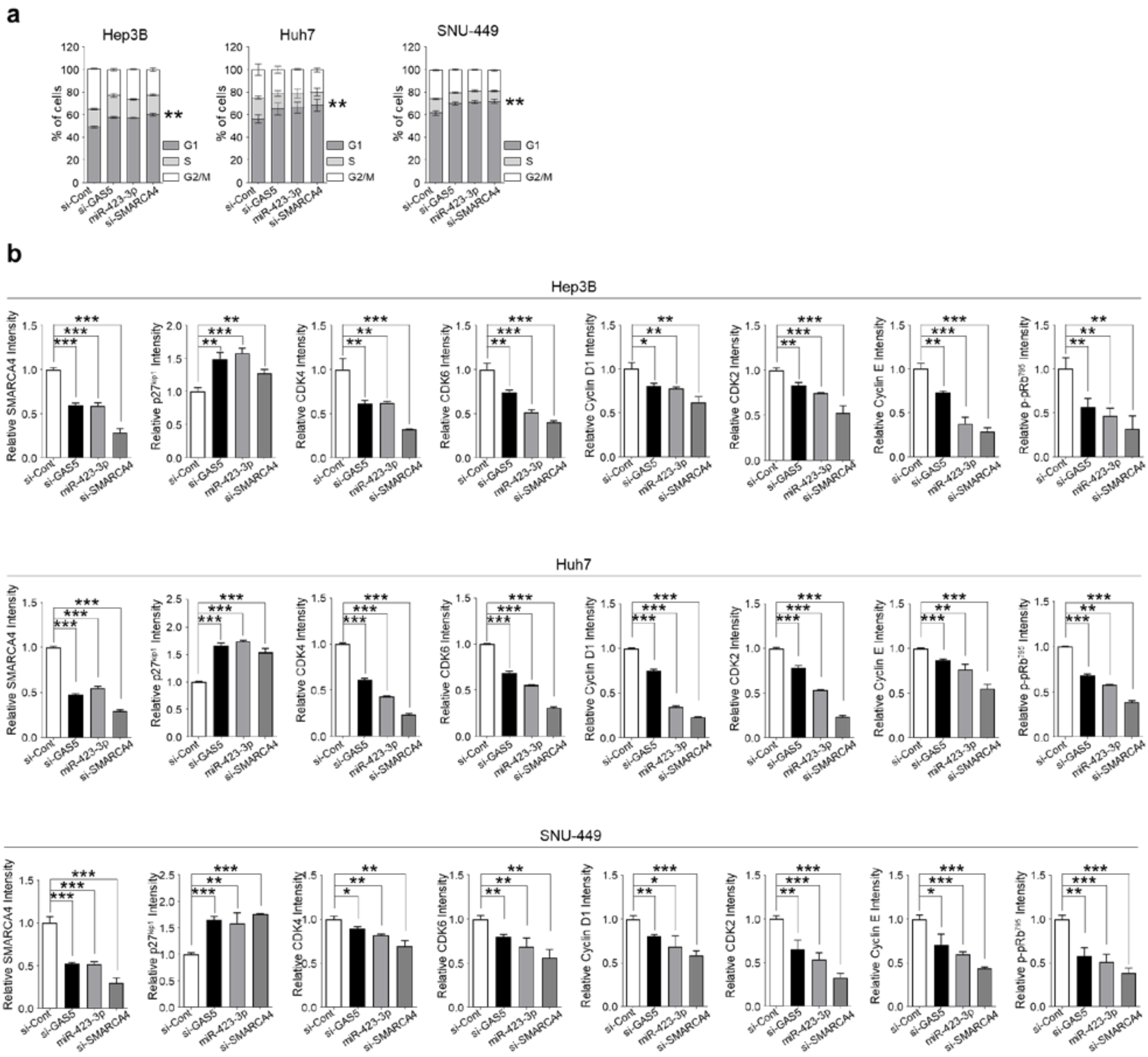

Supplementary Fig. 15 Knockout of either GAS5 or SMARCA4 induced G1/S arrest, exhibiting a phenotype similar to that observed with miR-423-3p overexpression in HCC cell lines. a The bar graph indicates the percentage at each cell cycle phase after transfections of si-Cont, si-GAS5, si-SMARCA4, and miR-423-3p mimic in Hep3B, Huh7, and SNU-449 HCC cells. **b** Densitometric analysis of cell cycle modulators protein expression in HCC cells following transfections corresponding to **(a)**. All data are shown as the mean ± SEM, *$p < 0.05$, **$p < 0.01$, ***$p < 0.001$; unpaired t test.

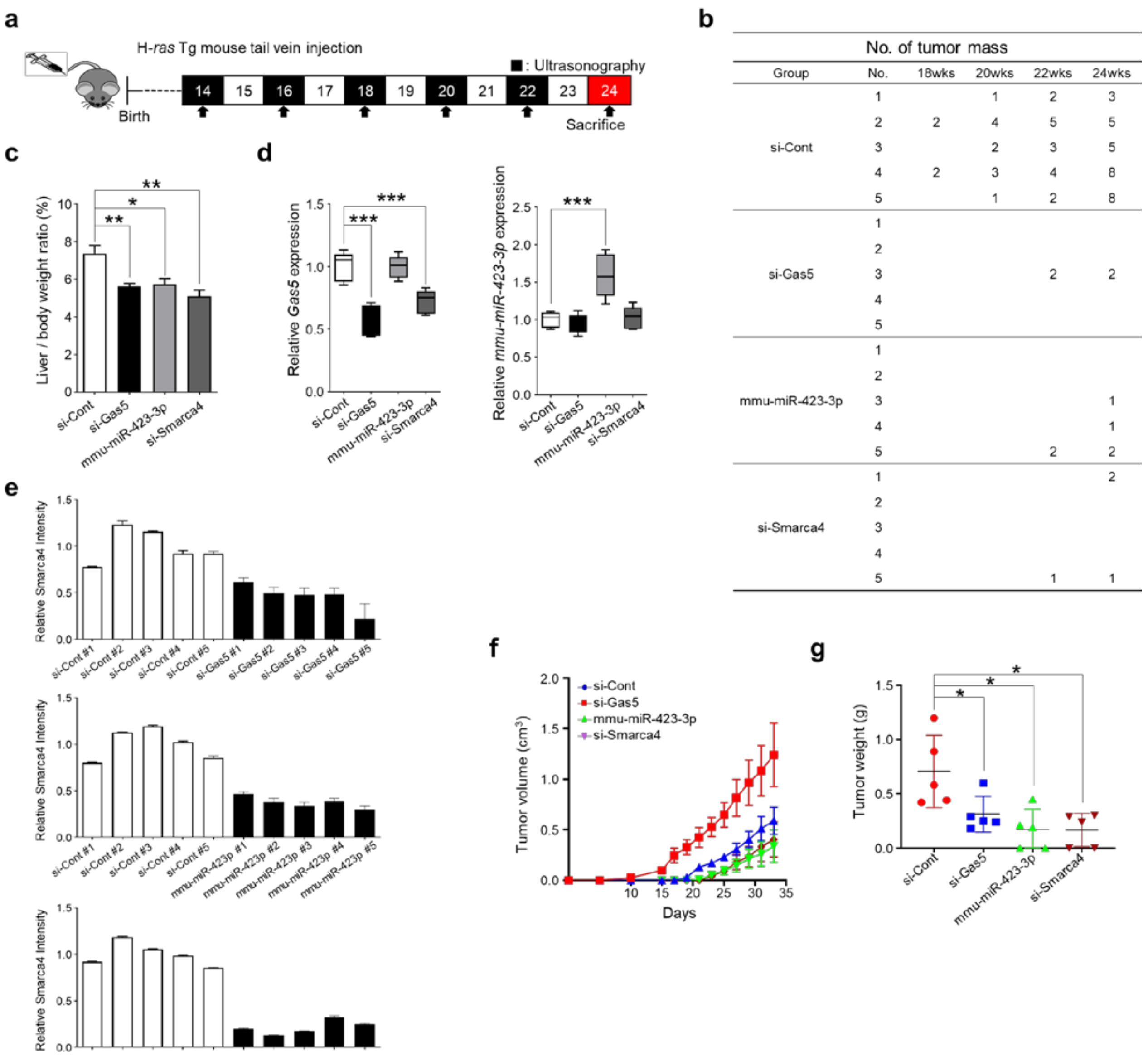

No. of tumor mass					
Group	No.	18wks	20wks	22wks	24wks
si-Cont	1		1	2	3
	2	2	4	5	5
	3		2	3	5
	4	2	3	4	8
	5		1	2	8
si-Gas5	1				
	2				
	3			2	2
	4				
	5				
mmu-miR-423-3p	1				
	2				
	3				1
	4				1
	5			2	2
si-Smarca4	1				2
	2				
	3				
	4				
	5			1	1

Supplementary Fig. 16 HCC progression is repressed by inhibition of sponge effect with GAS5-miR-423-3p-SMARCA4. **a** Time schedule of tail vein injection and ultrasonography into *Ras*-Tg mouse model. **b** The tumor mass number of each mouse at indicated weeks of age are listed in the table. **c** Bar charts show liver weight per body weight ratio in each group. **d** Quantitative RT-PCR analyses were performed for Gas5 and mmu-miR-423-3p expression. The Gapdh and U6 were used for loading control. **e** SMARCA4 protein expression intensity was quantified by densitometric analysis. **f** Tumor growth was measured by every three days in each group, and finally 33 days after injection. **g** Dot plots show tumor weight in each group. All data are shown as the mean ± SEM, *$p < 0.05$, **$p < 0.01$, ***$p < 0.001$; unpaired *t* test.

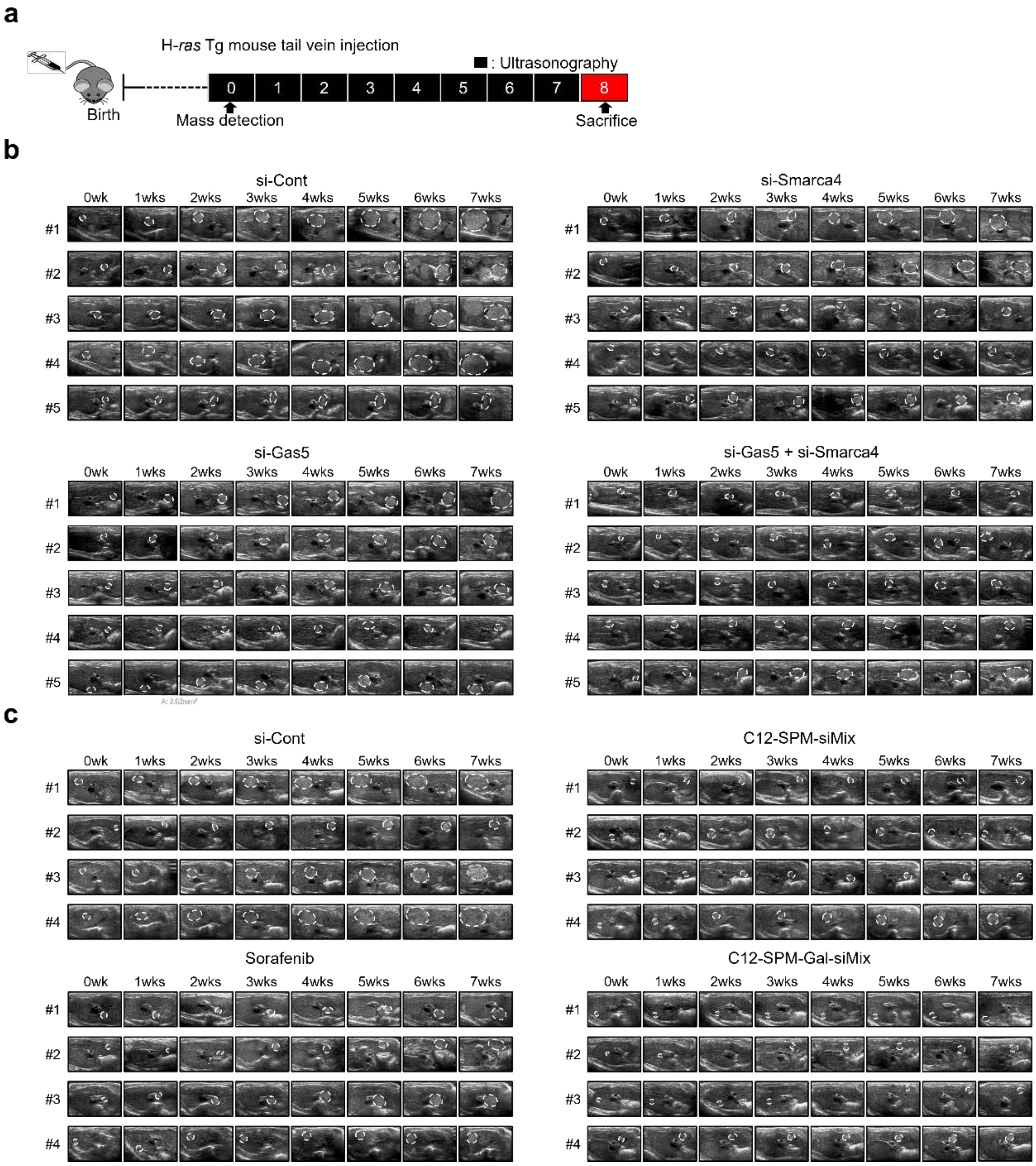

Supplementary Fig. 17 Ultrasonic images indicated that double knock-out of GAS5 and SMARCA4 significantly inhibit tumorigenesis in liver cancer models *in vivo*. a The schedule indicated tail vein injection (0; mass detection) for si-Cont, si-Gas5, si-Smarca4, si-Gas5+si-Smarca4 mix, sorafenib, C12-SPM-siRNA mix or C12-SPM-GAL-siRNA combination in *Ras*-Tg mouse models and sacrificed at eight weeks after injections. Ultrasonic images were observed every week. **b** Weekly ultrasonography images of Ras-Tg mice injected with si-Cont, si-Gas5, si-Smarca4, si-Gas5+si-Smarca4 mix. **c** Weekly ultrasonography images of *Ras*-Tg mice injected with si-Cont, siRNAs encapsulated liposomal nanoparticles (C12-SPM-siRNA mix or C12-SPM-GAL-siRNA mix), or sorafenib.

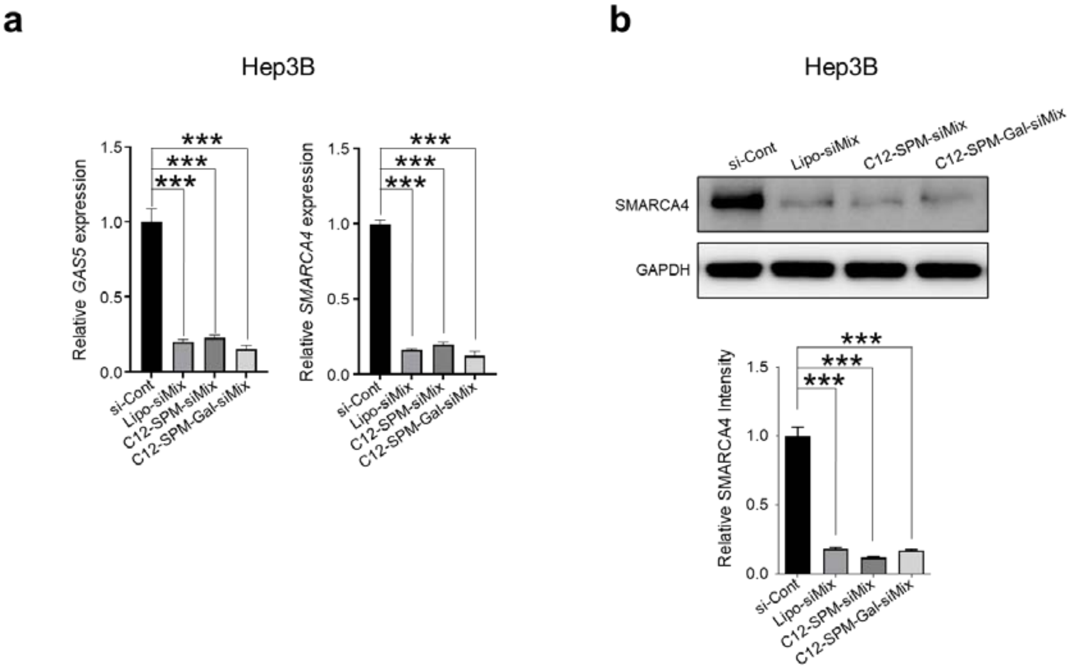

Supplementary Fig. 18 Knock-down efficiency using liposomal nanoparticles were evaluated with C12-SPM-siRNA mix or C12-SPM-GAL-siRNA mix *in vitro*. a SMARCA4 or GAS5 expression (qRT-PCR) was measured in Hep3B HCC cell lines, after transfected with si-Con, Lipo-siMix, C12-SPM-siRNA mix or C12-SPM-GAL-siRNA combination. **b** SMARCA4 protein expression (top) and quantification graph (bottom) after transfection in the Hep3B cell line, as in **(a).** All data are shown as the mean ± SEM, ***$p < 0.001$; unpaired t test.
